# Thermally Modulated Metasurface Sensor for Dynamic and Time‐Resolved Isolation of Extracellular Vesicles

**DOI:** 10.1002/adma.202522964

**Published:** 2026-05-08

**Authors:** Beyza Nur Kucuk, Eylul Gulsen Yilmaz, Yusuf Aslan, Emre Ece, Özgecan Erdem, Murat Alp Gungen, Bengi Özgün Öztürk, Fatih Inci

**Affiliations:** ^1^ UNAM—National Nanotechnology Research Center Bilkent University Ankara Turkey; ^2^ Institute of Materials Science and Nanotechnology Bilkent University Ankara Turkey; ^3^ Faculty of Science Department of Chemistry Hacettepe University Ankara Turkey

**Keywords:** extracellular vesicles (EVs), metasurface, plasmonic sensor, PNIPAM, smart polymer

## Abstract

Extracellular vesicles (EVs) are nanoscale lipid‐bilayered structures that mediate intercellular communications by transporting nucleic acids, proteins, and lipids across diverse biological fluids. Their diagnostic potential is immense, yet their heterogeneity poses persistent challenges for isolation and characterization, often leading to low yield, co‐isolation of contaminants, and vesicle damage. Here, we present a thermoresponsive polymer–integrated plasmonic metasurface sensor that enables spatiotemporally controlled, label‐free EV isolation. The metasurface, engineered by repurposing nanograted optical disks, was functionalized with poly(*N*‐isopropylacrylamide) (PNIPAM) and anti‐CD63 antibodies to achieve selective EV capture at physiological temperature and gentle release upon a minute thermal change near the polymer's lower critical solution temperature (∼35°C). Using MCF‐7 and HEK‐293–derived EVs as a proof‐of‐concept, the platform exhibited a dynamic detection range spanning three orders of magnitude. Release efficiency reached 87.03 ± 23.5%, while both nanoparticle tracking analysis (NTA) and fluorescent NTA (fNTA) revealed up to ∼100‐fold increase in EV purity relative to the ultrafiltration process. Electron microscopy and Western blotting confirmed preserved vesicle morphology and marker expression. By integrating thermoresponsive chemistry with a cost‐effective metasurface platform, this system offers a non‐destructive, portable, and real‐time solution for precise EV manipulation, advancing EV‐focused biosensing and point‐of‐care strategies for liquid biopsy applications in the future.

## Graphical abstract

A thermally adaptive plasmonic metasurface, functionalized with poly(*N*‐isopropylacrylamide) (PNIPAM) and anti‐CD63 antibodies, enables precise, on‐demand capture and release of extracellular vesicles (EVs). Delivering high recovery, improved purity, and intact vesicle structure, the platform offers a versatile, label‐free solution for real‐time EV isolation and biosensing.

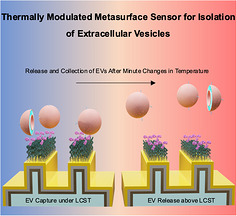

## 1 Introduction

Extracellular vesicles (EVs) are nanoscale lipid‐bilayered structures secreted by cells and present in diverse biological fluids, including blood, urine, saliva, cerebrospinal fluid, and milk [1, 2, 3]. They carry nucleic acids, proteins, lipids, and metabolites, mediating intercellular communications and maintaining cellular homeostasis [4]. EVs represent a heterogeneous and continuously evolving spectrum of secreted particles. While historically categorized into exosomes and ectosomes based on biogenesis, the International Society for Extracellular Vesicles (ISEV) now recommends the use of operational terminology such as small EVs (sEVs) and medium/large EVs (m/lEVs), unless specific biogenetic evidence is provided [5, 6]. In addition to classical membrane‐derived vesicles, emerging extracellular nanoparticle populations, including apoptotic bodies, migrasomes, large oncosomes, exomeres, and supermeres, further highlight the complexity of the EV landscape. This heterogeneity underpins their diverse functional roles but also poses analytical challenges, particularly for their isolation and characterization [7].

Numerous methods have been developed for EV isolation, exploiting differences in density, size, or surface markers [7, 8, 9, 10, 11, 12]. Differential ultracentrifugation, often considered the gold standard, separates EVs based on high centrifugal forces, yet suffers from co‐precipitation of contaminants, vesicle aggregation and damage, high equipment costs, and limited purity [13]. Density gradient and size‐exclusion chromatography methods improve purity but often yield highly diluted EVs [14]. Immunoaffinity approaches target specific tetraspanin proteins, achieving high selectivity but face scalability challenges and require harsh elution steps to recover intact vesicles [15]. Microfluidic‐based strategies offer small‐volume handling and integration with affinity or filtration methods [16, 17], yet many remain limited to size‐based separations. Overall, controlled, non‐destructive EV isolation remains an unmet need, as conventional approaches frequently compromise vesicle integrity and downstream molecular analyses. On the other hand, thermoresponsive polymers have emerged as promising tools for reversible biomolecule capture. Poly(*N*‐isopropylacrylamide) (PNIPAM), in particular, undergoes a sharp hydrophilic‐to‐hydrophobic transition near its lower critical solution temperature (LCST, ∼35°C), enabling precise thermal control over molecular interactions. While several studies have demonstrated the use of thermoresponsive polymer–integrated microfluidic chips [18] and sensors [19] for cell or protein biomarker isolation, platforms that combine high sensitivity with spatiotemporal control for EV isolation are still lacking.

**FIGURE 1:**
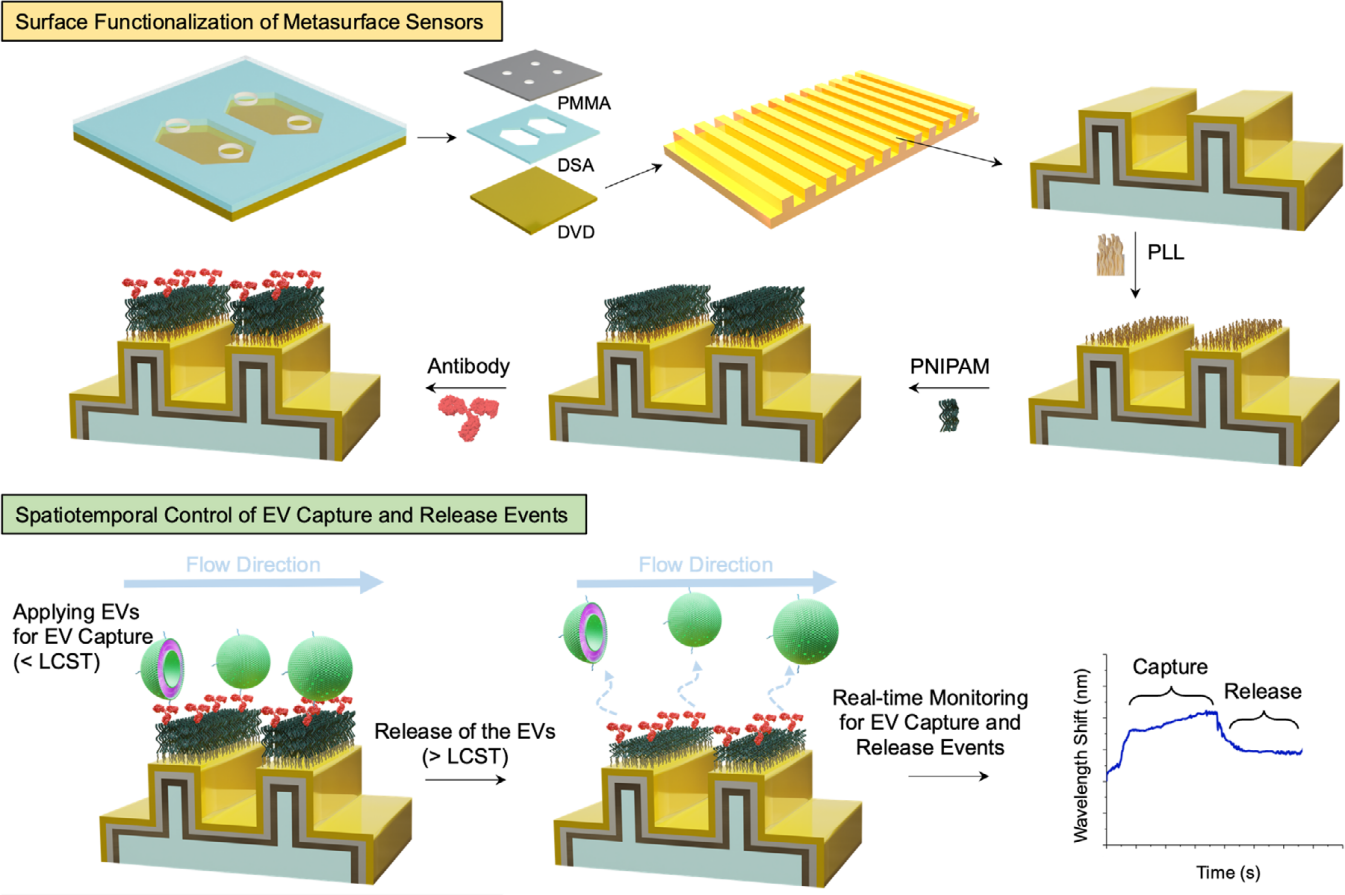
Schematic illustration of the thermoresponsive polymer–functionalized metasurface sensor for spatiotemporal capture and release of EVs. A microfluidic unit was first integrated with the metasurface sensor to enable controlled sampling. Prior to measurements, the sensor surface was sequentially functionalized with poly(L‐lysine) (PLL), thermoresponsive polymer PNIPAM, and EV specific antibodies. EVs were captured at room temperature (~27°C) and subsequently released upon thermal stimulation above the LCST of the polymer (∼35°C).

In this study, we present a thermoresponsive polymer–integrated metasurface sensor for spatiotemporal isolation of EVs. The sensors were initially coated with an adlayer molecule, followed by functionalization with PNIPAM and anti‐CD63 antibodies to enable specific, label‐free EV capture. Subsequently, these EVs were released through a minute temperature change, allowing their isolation for downstream characterization (Figure 1). To achieve precise spatiotemporal control of capture and release events, the metasurface sensor was designed by repurposing optical disks with inherent nanograting structures, significantly reducing fabrication cost and time. Real‐time changes throughout this process were managed through a portable system. As a proof of concept, EVs secreted by MCF‐7 breast cancer cells were utilized, and the platform demonstrated a dynamic detection range spanning three orders of magnitude. Release efficiency reached 87.03 ± 23.5% after surface blocking, confirming that passivation enhances specific EV release. Nanoparticle tracking analysis (NTA) and fluorescent NTA (fNTA) revealed a ∼100‐fold increase in EV purity compared to ultrafiltration methods (initial collection), while scanning electron microscopy (SEM), transmission electron microscopy (TEM), and Western blot analyses validated vesicle morphology and specific marker expression. Collectively, this platform overcomes key limitations of existing approaches by combining low‐cost, portability, real‐time monitoring, and high specificity, representing a promising tool for future EV‐based diagnostics in biomedicine.

## 2 Materials and Methods

### 2.1 Materials

*N*‐isopropylacrylamide (NIPAM), 2‐bromoisobutanoic acid *N*‐hydroxysuccinimide ester (NHS‐BiB), poly‐*L*‐lysine (PLL), copper(I) bromide (CuBr), methanol (≥99.9%), absolute ethanol (≥99.5%), acetone (≥99.5%), and isopropanol were all procured from Sigma–Aldrich (USA). Phosphate‐buffered saline (PBS) tablets were obtained from Biomatik (Canada), while tris(2‐dimethylaminoethyl)amine (Me6TREN) was sourced from Tokyo Chemical Industry (TCI, Japan). For metasurface fabrication, commercially available optical (DVD‐R) discs (HP, USA) were utilized as diffraction grating substrates. High‐purity gold (Au, 99.99%) and silver (Ag, 99.90%) pieces were supplied by the Istanbul Gold Refinery (Turkey). High‐purity titanium (Ti, 99.995%) pellets were supplied by NANOVAK (Turkey).  For human breast adenocarcinoma (MCF‐7) cell culture, Dulbecco's Modified Eagle Medium/Nutrient Mixture F‐12 (DMEM/F‐12) supplemented with L‐glutamine, sodium bicarbonate, and HEPES, along with fetal bovine serum (FBS), trypsin–EDTA (0.25%), and penicillin (10 000 U/mL)–streptomycin (10 000 µg/mL), were purchased from Thermo Fisher Scientific, Gibco (USA). For microfluidic chip fabrication, poly(methyl methacrylate) (PMMA) sheets were obtained from Sumitomo Chemical (Japan), and double‐sided adhesive (DSA) films were procured from 3M^TM^ (USA) and patterned via laser cutting. Polytetrafluoroethylene (PTFE) capillary tubing was acquired from Adtech (UK).

### 2.2 Cell Culture

MCF‐7 was cultured in Dulbecco's Modified Eagle Medium/Nutrient Mixture F‐12 (DMEM/F‐12) supplemented with 10% fetal bovine serum (FBS) and 1% penicillin–streptomycin. Cells were maintained in T75 culture flasks at 37°C in a humidified incubator with 5% CO_2_. The culture medium was refreshed every two days, and subculturing was performed at 80%–90% confluency using 0.25% trypsin–EDTA solution. The details for culturing human embryonic kidney (HEK‐293) cell line is stated in Supporting Information file.

### 2.3 EV Collection and Characterization

In the proof‐of‐concept experiments, MCF‐7 cells were employed, whereas HEK‐293 cells were utilized to evaluate the versatility of the platform and strategy. Accordingly, the detailed protocols for EV from MCF‐7 cells are stated in the manuscript, while those for EV isolation for HEK‐293 are provided in the Supporting Information. Briefly, MCF‐7 cells were seeded in T75 flasks and cultured for four days without medium replacement, as described previously [16]. On day 4, the conditioned medium containing secreted EVs was collected and transferred into 2 mL centrifuge tubes. The samples were centrifuged at 20 000 × *g* for 15 min at room temperature to remove cellular debris and apoptotic bodies, and the resulting supernatant was passed through 0.22 µm syringe filters to obtain a clarified EV‐containing medium. The purified medium was subsequently processed using a custom‐designed microfluidic ultrafiltration chip (20 × 40 mm), composed of five PMMA and five DSA layers, two polycarbonate membranes (200 and 50 nm pore sizes), and PTFE capillary tubing (Figure S1). Layers 1 and 2 contained the inlet and outlet structures, whereas layers 3–6 formed intermediate reservoirs for EV‐containing medium. Between layers 6 and 7, polycarbonate membranes were embedded to achieve sequential, size‐based isolation. EVs were first retained on the 200 nm membrane and subsequently enriched on the 50 nm membrane. Layers 8 and 9 defined the microchannel network directing flow from inlet to outlet, and layer 10 functioned as the structural base. Tubing connections were secured with epoxy, and the flow rate was controlled at 40 µL/min using syringe pumps. The collected EVs were characterized by NTA to determine their size distribution and concentration, and by Western blotting to confirm the presence of canonical EV surface markers (Supporting Information). Collected EV suspensions were aliquoted into 500 µL tubes and stored at –20°C for single use to minimize freeze–thaw degradation.

### 2.4 Fabrication of Metasurface Sensor

Commercially available, low‐cost optical discs were employed as templates for metasurface fabrication, as previously described [20]. The protective top layer of the discs was carefully peeled off to expose the underlying grating structure, which were subsequently cleaned in an ethanol:methanol (1:1, v/v) solution at room temperature. A brief etching process was then carried out in acetone:isopropanol (1:4, v/v) for 60 s, followed by thorough rinsing with distilled water and drying using an air gun. The treated substrates were sequentially coated with titanium (5 nm) as an adhesion layer, silver (30 nm) to impart plasmonic activity, and gold overlayer (15 nm) to synergistically enhance the plasmonic response, prevent potential silver oxidation, and improve long‐term stability. Metal deposition was performed by thermal evaporation under 10^−6^ Torr using a MiDAS PVD 3T system (VAKSIS, Turkey) (Figure S2). The fabricated metasurfaces were subsequently sectioned into 15 × 15 mm chips for integration with the microfluidic platform.

### 2.5 Fabrication of Microfluidic Platform for Sensors

Microfluidic chips were fabricated using PMMA (2 mm thickness) and DSA (50 µm thickness) layers. The device designs were generated using RDWorks software and patterned via laser cutting (LazerFix, Turkey). The PMMA layer was machined to form the inlet and outlet ports (40% of power and 40 mm/s of speed), while the DSA layer (15% of power and 100 mm/s of speed) defined the microchannel geometry and served as the bonding interface to the plasmonic metasurface. Prior to assembly, PMMA substrates were cleaned with 70% ethanol and thoroughly dried. The components were then sequentially aligned and laminated in the following order: metasurface, DSA, and PMMA. PTFE capillary tubing (15–20 cm in length) was then inserted into the inlet and outlet ports and secured using epoxy resin. Fluid flow through the microchannels was precisely controlled by syringe pumps (New Era Pump Systems Inc., USA) (Figure S3).

### 2.6 Synthesis of Thermoresponsive Polymer

Prior to atom transfer radical polymerization (ATRP), the NIPAM monomer was recrystallized in *n*‐hexane and thoroughly dried. ATRP was performed using NIPAM, CuBr, Me_6_TREN, and NHS‐BiB in a molar ratio of 100:1:1:1. Briefly, NIPAM (600 mg) was dissolved in deionized water (10 mL) within a round‐bottom Schlenk flask and stirred at 250 rpm for 10 min at 4°C. In a separate Schlenk flask, Me_6_TREN (12 mg) was dissolved in deionized water (4 mL). Both solutions were purged with nitrogen gas for 10 min under gentle stirring to remove dissolved oxygen. Subsequently, CuBr (7.63 mg), NHS‐BiB (14 mg), and the Me_6_TREN solution were sequentially introduced into the NIPAM solution, followed by an additional 30 min of nitrogen purging at 4°C. The polymerization reaction was then allowed to proceed at room temperature for 2 h. Upon completion, PNIPAM aggregates were collected by gentle heating with a hot air gun, transferred to a Petri dish, and dried at room temperature for 24 h. The dried PNIPAM was stored in a desiccated and dark environment until further use.

### 2.7 Surface Modification and Characterization of Sensors

Metal‐coated polycarbonate metasurfaces (15 × 15 mm) were first sterilized in 70% ethanol, air‐dried, and sequentially functionalized with PLL (0.1 mg/mL in PBS, overnight at 4°C), NHS‐terminated PNIPAM (2.5–10 mg/mL in distilled water, overnight at 4°C), anti‐CD63 antibody (50 µg/mL, 3 h at 4°C), and Bovine Serum Albumin (BSA) (5 mg/mL in PBS, 20 min at 4°C) for surface passivation. After each modification step, the substrates were gently rinsed with PBS and dried under a stream of nitrogen. Following functionalization, the sensors were integrated into the microfluidic platform for real‐time plasmonic measurements of EV capture and thermally induced release. Surface characterization was conducted using contact angle goniometry, X‐ray photoelectron spectroscopy (XPS), SEM, and laser scanning microscopy. Detailed characterization protocols and analysis parameters are provided in the Supporting Information.

### 2.8 Real‐time Monitoring and Detection of EV Capture and Release on Metasurface Sensor

A portable optical sensing system was employed for real‐time monitoring of EV capture and thermally induced release (Figure S4A,B). The setup comprised a compact spectrometer (Thorlabs, CCS175/M) and a fiber‐coupled broadband light source (Thorlabs, OSL2) operating in the visible range, both enclosed within a custom 3D‐printed housing for optical and environmental stability. The incident light was directed onto the plasmonic metasurface via a series of lenses, a linear polarizer, and a beam splitter, while the reflected spectra were collected by the spectrometer and analyzed using the Thorlabs OSA software. The spectral shifts were processed through a MATLAB‐based graphical user interface (GUI). The final data were plotted as wavelength (nm) versus time (s). Sample flow was maintained at 10 µL/min using a syringe pump. A standard measurement cycle for capture events was followed: PBS (5 min) for baseline stabilization, EV sample injection (45 min, association phase), and PBS washing (5 min, dissociation phase). Binding interactions were quantified based on wavelength shifts relative to the initial baseline.

### 2.9 Real‐time Temperature Modulation on Sensors

A custom heating system was developed to regulate the temperature of the sensor using a Proportional–Integral–Derivative (PID) control algorithm implemented on an Arduino UNO microcontroller. The system employed a repurposed 3D printer heating pad as the heat source, with temperature feedback provided by a thermistor sensor placed on the heating pad surface. Temperature data were continuously monitored by the Arduino, which adjusted the power supplied to the heating pad via pulse‐width modulation (PWM) through a motor driver, powered by a DC power supply. This setup enabled stable and precise temperature control during experiments.

For thermal modulation, the printed circuit board (PCB) heating unit (Figure S4A,B) was positioned beneath the sensor, and temperature was regulated by an Arduino‐assisted control system (Figure S4C) programmed via a Python‐based interface. EV capture was performed at 27°C, followed by PBS washing to remove unbound particles. For controlled release, the temperature was elevated to 35°C while maintaining a constant flow rate of 10 µL/min for 45 min. The wavelength shifts were recorded before and after heating were compared to determine EV release efficiency.

## 3 Results and Discussion

### 3.1 Cell Studies

In this study, MCF‐7 breast cancer cells were cultured under the conditions described in the earlier section. On the fourth day of incubation—previously reported as the optimal timepoint for EV collection [16]—the culture medium was harvested, and the cells were imaged using an inverted fluorescence microscope. To assess cell viability, calcein AM, a live‐cell indicator that produces green fluorescence, and ethidium homodimer, a dead‐cell indicator that fluoresces red, were employed. In addition, bright‐field and Hoechst 33342 nuclear staining images were acquired to evaluate cell morphology and confluency. The bright‐field image (Figure S5A) shows that MCF‐7 cells adhered uniformly to the culture surface, forming a confluent monolayer essential for healthy proliferation. The observed epithelial‐like morphology was consistent with previous reports, confirming that the culture conditions were optimal for the secretion of physiologically relevant EVs [21, 22]. Live‐cell staining with calcein AM revealed extensive green fluorescence (Figure S5B), whereas only a few red‐stained cells were observed following ethidium homodimer labeling (Figure S5C). The merged live/dead image (Figure S5D) demonstrated that the vast majority of the cells remained viable on the fourth day without medium exchange—an important criterion for unperturbed EV secretion. Hoechst 33342 nuclear staining (Figure S5E) provided clear visualization of the nuclei and confirmed the confluency of the culture, complementing the bright‐field observations. To further validate the cancer‐specific phenotype of the cultured cells, immunohistochemistry (IHC) staining was performed to detect key epithelial and tumor‐associated markers: cytokeratin‐19 (CK‐19), a major cytoskeletal intermediate filament protein, whose dysregulated expression is often associated with oncogenic transformation [23]; and epithelial cell adhesion molecule (EpCAM, CD326), a transmembrane glycoprotein highly expressed in epithelial‐derived carcinomas [24].

Hoechst 33342 staining (Figure S5F) was used to visualize the nuclei under the DAPI channel (ex/em: 350/461 nm). Alexa Fluor 647–conjugated anti‐EpCAM antibody (Figure S5G) was imaged using the RFP channel (ex/em 650/671 nm), revealing strong red fluorescence along the cell membranes, consistent with high EpCAM expression. FITC‐labeled anti‐CK‐19 antibody (Figure S5H) was visualized using the GFP channel (ex/em: 495/519 nm), highlighting the cytoskeletal filaments distributed across the cytoplasm. The strong and spatially distinct expression of CK‐19 and EpCAM confirmed the epithelial and cancerous phenotype of the MCF‐7 cell line (Figure S5I), validating the cell model for subsequent EV isolation experiments.

### 3.2 EV Isolation via Ultrafiltration Chip and Characterization Studies

In accordance with the Minimal Information for Studies of Extracellular Vesicles (MISEV) 2018 guidelines [25], the collected and released EVs were rigorously characterized using four complementary analytical approaches: Western blotting for protein marker validation, NTA for size distribution, fNTA for marker‐specific confirmation, and electron microscopy for morphological assessment. EVs isolated using the ultrafiltration chip were characterized by SEM and NTA to serve as a reference for comparison with those subsequently isolated through thermoresponsive polymer‐modified sensor capture and release in real‐time. SEM was employed to assess the morphology and organization of micron‐ and nanoscale structures. As expected for lipid bilayer–encapsulated vesicles, EVs exhibited a predominantly spherical morphology [26]. In Figure S6A, uniformly distributed, spherical EVs were observed, although some aggregation occurred during fixation. These aggregates led to localized roughness and uneven Au/Pd coating, resulting in brighter regions due to electron accumulation. A higher‐magnification image (Figure S6B) clearly reveals EVs with homogeneous, round‐shaped morphology, free of contaminants (e.g., larger bioparticles) and without signs of rupture or deformation. The size distribution and concentration of EVs isolated by the ultrafiltration chip were further analyzed using NTA. The average particle size was determined as 137.7 ± 3.6 nm with a modal size of 94.4 ± 4.7 nm, consistent with the pore dimensions of the filters (200 and 50 nm). The particle concentration was measured as 1.01 × 10^10^ ± 5.23 × 10^8^ particles/mL (Figure S6C). To assess the purity of the isolated EVs, an immunostaining protocol was performed targeting the tetraspanin CD63, a canonical EV surface marker. Alexa Fluor 488–conjugated anti‐CD63 antibody (ex/em: 490/525 nm) was used, and fNTA was performed using a 488 nm laser on the instrument. Prior to measurement, EVs were diluted 1:50 in PBS, mixed with the fluorescent antibody, and incubated for 30 min in the dark at room temperature. Following immunostaining, the mean and mode particle sizes decreased to 75.2 ± 13.6 nm and 54.1 ± 20.2 nm, respectively, with a corresponding particle concentration of 1.98 × 10^6^ ± 1.71 × 10^5^ particles/mL—approximately 10^4^‐fold lower than the unlabeled sample, confirming selective identification of CD63^+^ EVs (Figure S6D).

### 3.3 Synthesis, Chemical and Thermal Characterization of Thermoresponsive Polymer

**FIGURE 2:**
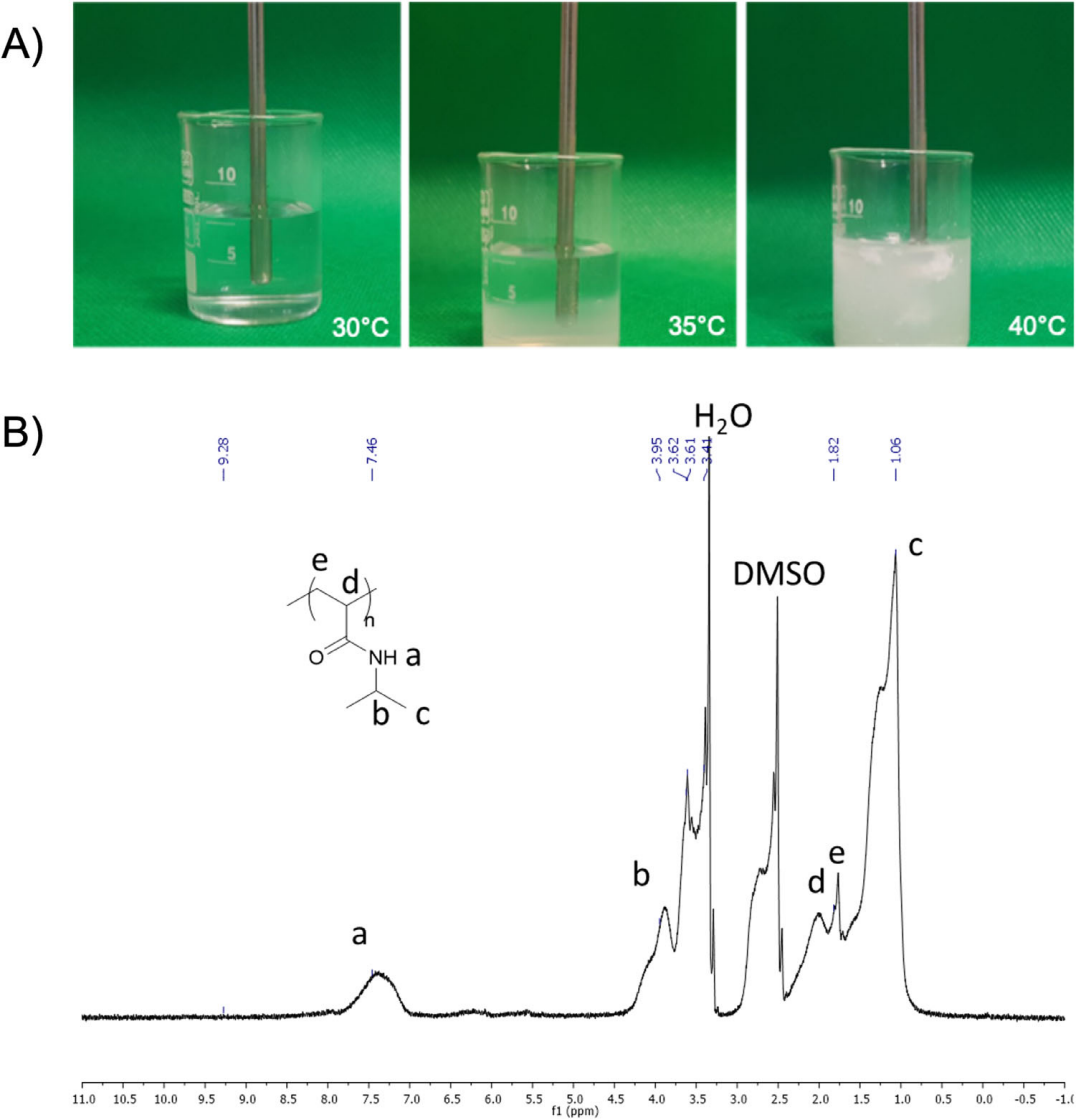
Characterization of thermoresponsive polymer (PNIPAM). (A) Visual demonstration of the PNIPAM solution transitioning from hydrophilic to hydrophobic states at 30°C (below LCST), 35°C (near LCST), and 40°C (above LCST). (B) ^1^H NMR spectrum of synthesized PNIPAM, confirming successful polymerization and chemical composition.

To enable spatiotemporal control over EV isolation, a thermoresponsive polymer layer was integrated onto the metasurface sensor. The design rationale was to exploit the LCST behavior of the polymer, allowing reversible EV detachment from the sensor surface upon temperature elevation beyond the LCST. The thermoresponsive polymer (PNIPAM) was synthesized via ATRP in a liquid‐phase system. To ensure the removal of CuBr catalyst residues, the synthesized PNIPAM solution was gently heated to induce the formation of hydrophobic PNIPAM aggregates. These aggregates were subsequently transferred onto a clean Petri dish, air‐dried into solid form, and later redissolved in distilled water at the desired concentration for further studies. As described in the literature, PNIPAM is known to undergo a reversible phase transition from a hydrophilic to a hydrophobic state around its LCST, typically reported between 32°C and 35°C [27]. To experimentally validate this transition, a 10 mg/mL of PNIPAM solution in distilled water was prepared and gradually heated on a hot plate while continuously monitoring the temperature using a probe immersed in the sample (Figure 2A). At 30°C, below the LCST, the solution remained transparent, indicating complete solubility of the polymer in water without any visible aggregation. Upon increasing the temperature to 35°C, the solution began to exhibit turbidity, signifying the onset of hydrophobic interactions and polymer aggregation—particularly noticeable near the bottom of the vial, closest to the heat source. At 40°C, the transition was complete, and the solution turned opaque white, confirming the formation of hydrophobic PNIPAM aggregates. These observations validated the thermoresponsive nature of the synthesized polymer and established 35°C as the precise transition temperature. This temperature was subsequently selected as the operational release condition for the next plasmonic experiments, as it is sufficiently mild to preserve the structural integrity of biological specimens such as EVs.

Since we planned to enable subsequent bioconjugation with the thermoresponsive polymer, PNIPAM chains were functionalized with NHS ester groups, which readily react with primary amines abundant in biomolecules such as proteins and antibodies [28]. For this purpose, an NHS‐terminated ATRP initiator (NHS–BiB) was employed during polymerization, resulting in PNIPAM chains bearing terminal NHS functionalities. The successful incorporation of NHS groups and the structural integrity of the PNIPAM backbone were confirmed by nuclear magnetic resonance (NMR) spectroscopy. The spectra were recorded in deuterated dimethyl sulfoxide (DMSO‐d_6_), with chemical shifts reported in parts per million (ppm) relative to the residual DMSO signal (Figure 2B, Table S1). The characteristic resonances confirmed the formation of NHS‐terminated PNIPAM and validated the efficiency of the ATRP synthesis route. The ^1^H‐NMR spectrum exhibited characteristic PNIPAM signals: amide (─NH) protons at ∼7.46 ppm, methine protons of the polymer backbone at 3.95–3.44 ppm, isopropyl methyl protons at 1.82–1.06 ppm, and methylene protons at ∼0.9–1.2 ppm. Additionally, a minor resonance shoulder observed at 2.8–3.0 ppm corresponded to the succinimide moiety, confirming the successful incorporation of NHS groups. These spectral features collectively validated the efficient synthesis of NHS‐terminated PNIPAM suitable for subsequent antibody conjugation and thermoresponsive biosensing applications.

### 3.4 Surface Wettability, Topography, and Chemical Characterization of Functionalized Metasurface Sensors

**FIGURE 3:**
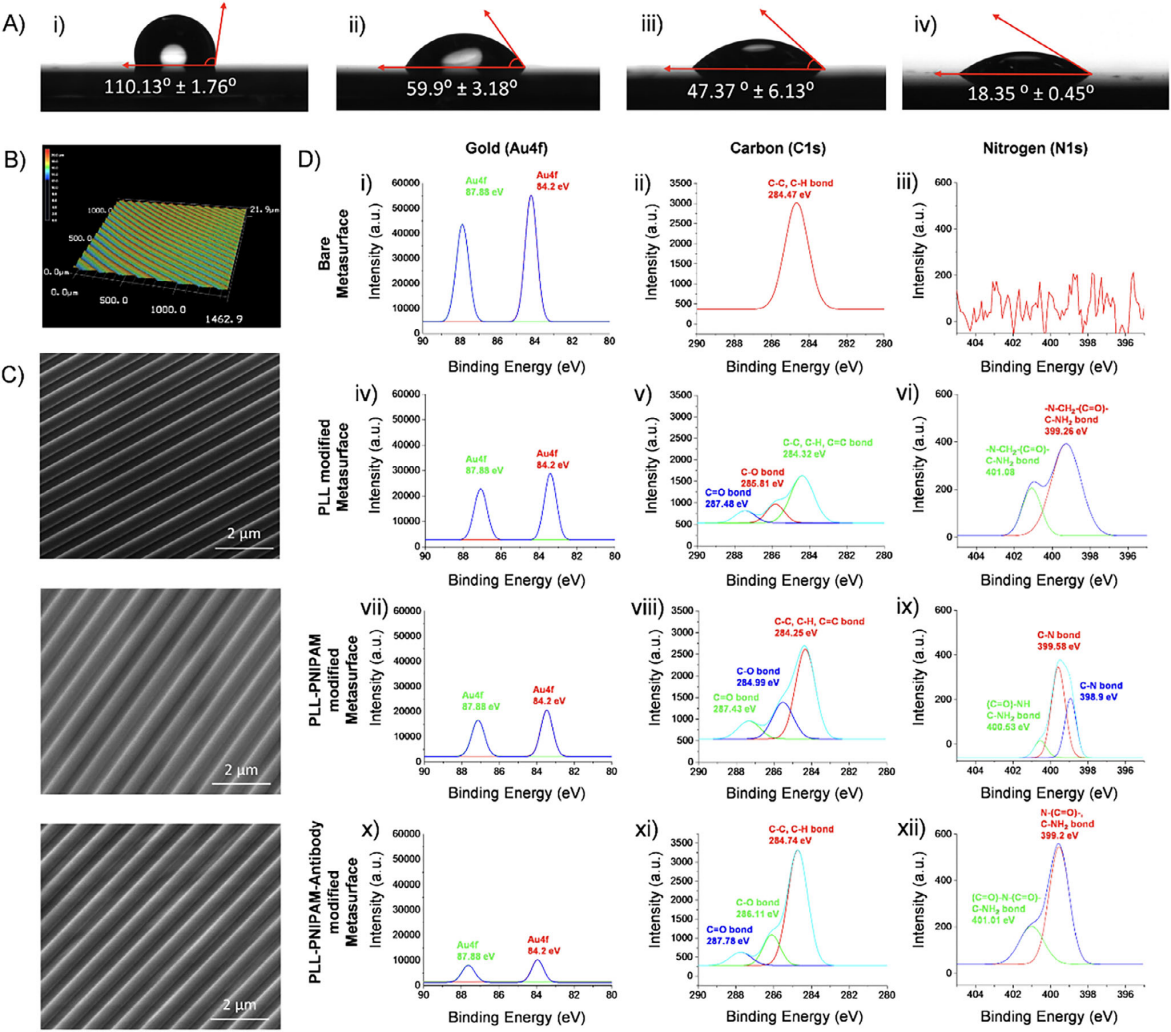
Characterization of metasurface sensors. (A) Contact angle measurements for (i) bare metasurface, (ii) PLL‐modified surface, (iii) PLL and PNIPAM–modified surface, and (iv) PLL, PNIPAM, and anti‐CD63 antibody–decorated sensor, illustrating the cumulative increase in hydrophilicity. (B) Laser scanning confocal microscopy image of the PLL and PNIPAM–modified sensor showing preserved grating topology after the surface chemistry approach. (C) SEM images of the bare metasurface sensor (top), PLL‐coated sensor (middle), and PLL and PNIPAM–modified sensor (bottom), confirming uniform surface coverage without structural disruption. (D) XPS spectra of bare and modified sensors (bare, PLL, PLL‐PNIPAM, and PLL‐PNIPAM‐antibody from top to bottom) for Au4f, C1s, and N1s, validating sequential chemical modifications.

We decorated the metasurface sensors with PLL as an adlayer and a thermoresponsive polymer layer for spatiotemporal control of capture and release events for EV isolation. In this scenario, sequential surface modifications of the sensor were evaluated using a combination of wettability, topography, and chemical analyses. The surface wettability of the metasurface sensors was evaluated through contact angle analysis to assess its hydrophilic–hydrophobic transition during surface modifications—a property directly influenced by surface energy. Herein, four surface configurations were examined sequentially: (i) the bare metasurface, (ii) 0.1 mg/mL of PLL‐modified metasurface, (iii) 0.1 mg/mL of PLL and 5 mg/mL of PNIPAM co‐modified metasurface, and (iv) 0.1 mg/mL of PLL, 5 mg/mL of PNIPAM, and 50 µg/mL of anti‐CD63 antibody functionalized metasurface (Figure 3A). The bare metasurface exhibited a contact angle of 110.13° ± 1.76°, characteristic of a hydrophobic surface with low surface energy. Following PLL modification, the contact angle decreased substantially to 59.9° ± 3.18°, indicating enhanced hydrophilicity due to the introduction of amine and hydroxyl groups. Further modification with PNIPAM reduced the contact angle to 47.37° ± 6.13°, consistent with the presence of the amide‐rich polymer layer. Upon immobilization of the anti‐CD63 antibody, the contact angle further decreased to 18.35° ± 0.45°, confirming the increasing hydrophilic nature of the surface following each successive modification. Collectively, these results demonstrated a progressive enhancement in surface hydrophilicity, supporting the successful sequential functionalization of the plasmonic metasurface.

The underlying grating structure of the metasurface is vital for sustaining surface plasmon (SP) coupling and generating measurable plasmonic responses during real‐time analyte binding and release [29]. Therefore, preserving the periodic grating topology throughout metal deposition and subsequent surface modifications was essential. To verify structural integrity, laser scanning confocal microscopy (Keyence VKX‐100) and SEM analyses were conducted following polymer modification (PLL and PNIPAM) (Figure 3B,C). Laser microscopy confirmed that the grating periodicity was retained (Figure 3B), while SEM imaging revealed well‐preserved topographies for the bare (Figure 3C), PLL‐modified, and PLL–PNIPAM‐modified metasurfaces. These findings confirmed that both metal coating and subsequent surface chemistry treatments did not disrupt the nanoscale grating morphology, ensuring reliable plasmonic signal transduction for real‐time EV capture and release events.

The chemical composition of the modified surfaces was further examined using XPS analysis. Spectra corresponding to Au4f, C1s, and N1s regions were acquired for bare, PLL‐modified, PLL–PNIPAM‐modified, and PLL–PNIPAM–anti‐CD63 antibody‐modified surfaces (Figure 3D). In all samples, Au4f peaks appeared at 87.88 and 84.2 eV, confirming the presence of the gold layer. Although peak intensities gradually decreased after each modification due to surface coverage, no significant peak shifts were detected, indicating that the underlying gold film remained chemically stable. Analysis of the C1s spectra revealed a clear evolution in surface chemistry. The bare gold surface exhibited only a weak contamination peak near 284.3 eV, consistent with adventitious carbon (Figure 3D‐ii). Following PLL modification (Figure 3D‐v), dominant peaks at 284.32 eV (C─C, C─H, C═C) were observed, along with moderate peaks at 285.81 and 287.48 eV corresponding to C─O and C═O bonds from the PLL backbone. Upon PNIPAM coating (Figure 3D‐viii), the peak at 284.25 eV intensified, reflecting the PNIPAM backbone, while the peaks at ∼281.0 and 287.43 eV also increased, confirming the incorporation of amide groups. After anti‐CD63 antibody immobilization (Figure 3D‐xi), a slight reduction in the 287.7 eV (C═O) peak intensity was detected, indicative of amide bond formation. The N1s spectra provided complementary evidence for successful surface functionalization. The bare surface exhibited a negligible N1s signal (Figure 3D‐iii). After PLL deposition (Figure 3D‐vi), distinct peaks appeared at 399.26 eV (amine, ─NH_2_) and 401.08 eV (amide, HN─C═O─), confirming the presence of nitrogen‐containing functionalities. Following PNIPAM modification (Figure 3D‐ix), additional peaks emerged at 398.9 eV (secondary amines from the isopropyl group) and 400.53 eV (amide or imide bonds, N─(C═O)─). The amine‐related signal at 399.58 eV corresponded to nitrogen in the PNIPAM backbone. Finally, upon antibody conjugation (Figure 3D‐xii), an increased intensity at 399.5 eV was observed, reflecting the conversion of NHS esters into stable amide bonds. Collectively, XPS results corroborated the stepwise surface functionalization, confirming the successful attachment of PLL, PNIPAM, and anti‐CD63 antibody layers without compromising the structural or chemical integrity of the plasmonic metasurface sensor.

### 3.5 Controlling Thermal Activity on the Metasurface Sensors

**FIGURE 4:**
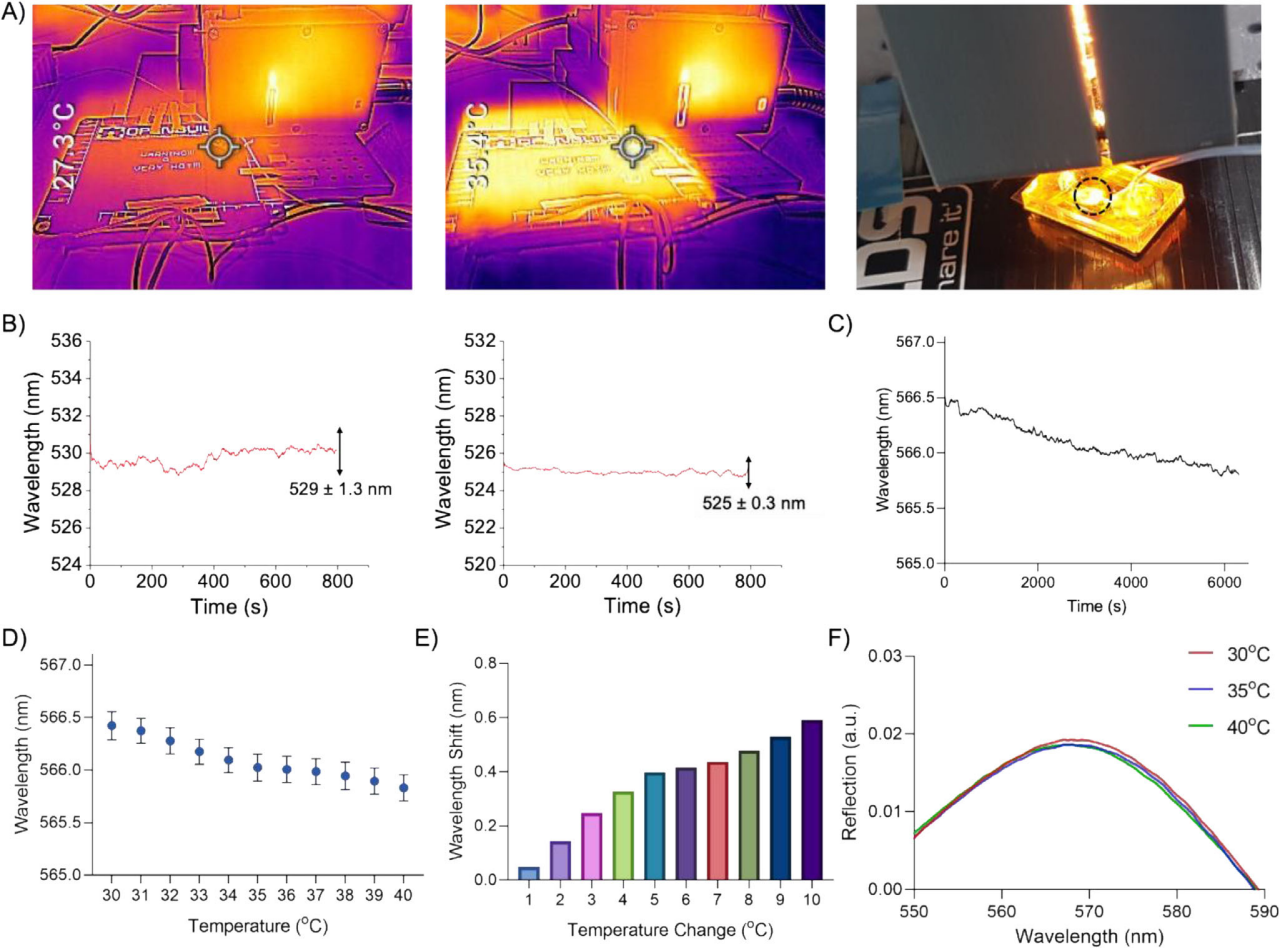
Optimization of thermoresponsive polymer (PNIPAM) concentration and temperature response for real‐time plasmonic sensing. (A) Thermal camera images captured at different stages of the experiment: capture at room temperature (∼27°C, below the LCST of PNIPAM) (left), release at elevated temperature (∼35°C, near the LCST) (middle), and PNIPAM aggregation within the microchannel at 45°C (right). (B) Real‐time plasmonic sensing of PNIPAM concentration after PLL modification: 10 mg/mL (left, *n* = 3) and 5 mg/mL (right, *n* = 3). (C–F) Temperature‐dependent refractive index changes assessed via plasmonic measurements: real‐time plasmonic signal, wavelength shifts with stepwise temperature increase (*n* = 600 datapoints were measured and analyzed for each temperature), quantified wavelength shift values, and reflection spectra recorded at 30, 35, and 40°C.

A custom‐designed PCB heater was integrated beneath the plasmonic metasurface sensor to achieve precise thermal control during EV capture and release events. The thickness of the PCB heater was measured, and the optical setup was accordingly adjusted to maintain the optimal focal distance of the incident light on the chip surface. To ensure experimental consistency under varying ambient conditions, the heater was initially set to 27°C—below the LCST of PNIPAM—for capture events. The temperature distribution across the sensor was verified using a thermal imaging camera (Figure 4A). As confirmed in previous PNIPAM characterizations (Figure 4A (left)), 27°C falls within the hydrophilic regime of PNIPAM, favoring EV capture on the functionalized surface. Subsequently, the temperature was increased to 35°C, corresponding to the hydrophilic‐to‐hydrophobic transition range of PNIPAM (Figure 4A (middle)). The uniformity and stability of this transition temperature were validated by a thermal camera (Figure 4A). Upon further heating to 45°C, visible aggregation of PNIPAM was observed on the sensor, forming solid‐like hydrophobic domains (Figure 4A (right)). This phenomenon further confirmed the successful polymer modification; yet, such aggregation was found to disrupt laminar flow, induce turbulence, and potentially interfere with plasmonic signal stability. To prevent such effects and maintain reproducible real‐time sensing performance, the release temperature was optimized and fixed near the phase transition threshold (35°C–37°C).

### 3.6 Optimization of Thermoresponsive Polymer Concentration and Temperature Response for Real‐Time Plasmonic Sensing

To establish optimal polymer coating conditions for real‐time analysis, the concentration of PNIPAM applied to the sensors was systematically optimized. A range of PNIPAM concentrations—10, 5, and 2.5 mg/mL—dissolved homogeneously in distilled water were evaluated for their effect on the plasmonic response (Figure 4B). Literature reports indicate that PNIPAM concentration significantly influences polymer conformation and surface uniformity [30, 31]. Once analyzing our data with triplicate measurements, at a PNIPAM concentration of 10 mg/mL, the resonance peak of metasurface sensors appeared near 529 nm; however, the signal exhibited considerable noise, with a standard deviation of 1.3 nm (Figure 4B (left)). Such high fluctuations obscure subtle binding events between the immobilized anti‐CD63 antibodies and target EVs, reducing the signal‐to‐noise ratio and limiting sensitivity. When the concentration was reduced to 5 mg/mL, the resonance peak shifted slightly to 525 nm, while the standard deviation decreased markedly to 0.3 nm—an acceptable noise level for reliable detection (Figure 4B (right)). Conversely, at 2.5 mg/mL, the polymer was not able to form a stable coating and no distinct PNIPAM response was observed at elevated temperatures, indicating insufficient surface coverage. Based on these findings, a PNIPAM concentration of 5 mg/mL was selected as optimal for subsequent capture–release events, balancing coating stability and signal fidelity.

Given the thermoresponsive nature of PNIPAM, the influence of temperature on the plasmonic signal was further examined using a PLL–PNIPAM–anti‐CD63 functionalized sensor (Figure 4C–F). In the process, PBS was continuously perfused through the microchannel of the sensors at a flow rate of 10 µL/min while the temperature was gradually increased from 30°C to 40°C in 1°C increments. For each temperature point, real‐time spectra were recorded for 10 min, and 600 data point measurements were analyzed (Figure 4C). The resonance wavelength exhibited a gradual red‐shift as temperature increased, consistent with previous reports of temperature‐dependent refractive index variations in PNIPAM systems [32].

To quantitatively assess this effect, the average wavelength and standard deviation of the final 500 data points at each temperature were analyzed (Figure 4D). The temperature‐induced spectral shift reached approximately 0.58 nm over a 10°C increase, confirming the sensitivity of the system to minor refractive index fluctuations (Figure 4E). Importantly, reflection spectra recorded at three key temperatures—30°C (<LCST), 35°C (transition point), and 40°C (>LCST)—revealed no irreversible distortions or degradation in signal quality, verifying the thermal stability of the metasurface (Figure 4F). To minimize the confounding influence of temperature‐driven wavelength drift during EV release events, an additional temperature stabilization step was incorporated. Following the release phase at elevated temperature (35°C–37°C), the system was cooled back to the capture temperature (27°C). The resonance wavelength before and after this thermal cycling was compared to quantify EV capture and release efficiency accurately.

Before implementing the PLL–PNIPAM–antibody system for EV capture, alternative surface modification chemistries were investigated to identify the most effective functionalization route. Two common linker strategies were tested (Figure S7): (i) 3‐mercaptopropyl‐N‐hydroxysuccinimide ester (3‐MNHS) and (ii) 3‐aminopropyltriethoxysilane (APTES). 3‐MNHS is a heterobifunctional crosslinker bearing a thiol (─SH) group capable of binding gold surfaces via Au–S interactions and an NHS ester group reactive toward primary amines, thereby serving as a potential intermediate for PNIPAM immobilization. APTES, by contrast, contains a silane moiety that reacts with hydroxylated surfaces to form siloxane bonds and presents terminal amine groups suitable for NHS–PNIPAM and antibody conjugation. Both chemistries were applied on separate sensors and assessed for EV capture efficiency relative to PLL–PNIPAM. For each condition, EV capture experiments were conducted using an EV suspension at 10^9^ particles/mL. Each experiment consisted of three sequential steps: (I) baseline measurement with PBS for 5 min at 10 µL/min, (II) EV introduction for 45 min at 10 µL/min, and (III) post‐capture washing with PBS for 5 min at the same flow rate. The net resonance wavelength shift after washing was used to assess the EV binding. As depicted in Figure S7A,B, 3‐MNHS‐functionalized surfaces exhibited wavelength shifts of 0.18 and 0.20 nm, yielding an average response of 0.19 ± 0.10 nm. Similarly, APTES‐treated surfaces (Figure S7C,D) produced minor shifts of 0.01 and 0.32 nm (average 0.165 ± 0.155 nm). Despite the relatively high EV concentration, both modification strategies generated negligible spectral responses—comparable to the baseline noise of the sensor. Consequently, neither APTES nor 3‐MNHS chemistries were deemed suitable for sensitive EV capture and release assays, and the PLL–PNIPAM surface modification was selected as the optimal platform for subsequent experiments.

### 3.7 Temperature‐Controlled Capture and Release of EVs on Metasurface Sensors

**FIGURE 5:**
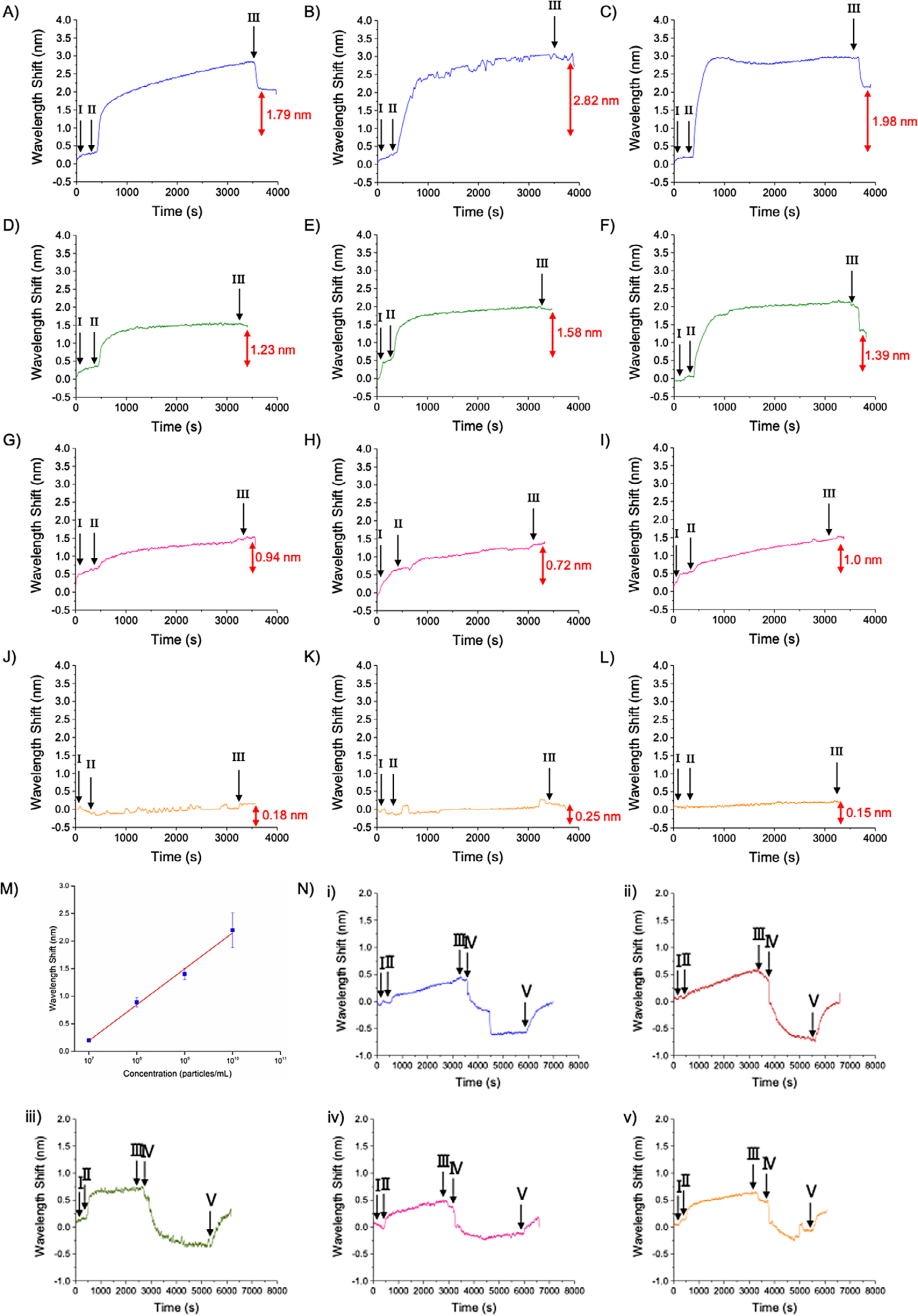
Real‐time plasmonic monitoring of EV capture and thermally triggered release on PLL–PNIPAM–anti‐CD63 antibody–modified sensors. Triplicate measurements of EV capture and release at varying concentrations: (A–C) 10^10^ (*n* = 3), (D–F) 10^9^ (*n* = 3), (G‐I) 10^8^ (*n* = 3), and (J–L) 10^7^ particles/mL (*n* = 3). (M) Mean wavelength shifts with standard deviation (error bars); *n* = 5 for each concentration, correlation analysis yielded Pearson Coefficient r = 0.94 and R^2^ = 0.89. (N) Representative wavelength shifts from five replicate experiments with BSA surface blocking.

Building upon the optimization studies, the metasurface sensors were functionalized sequentially with 0.1 mg/mL of PLL, 5 mg/mL of PNIPAM, and 50 µg/mL of anti‐CD63 antibody. The capture temperature was maintained at 27°C, below the LCST of PNIPAM, to ensure its hydrophilic state. The highest EV concentration obtained using the ultrafiltration chip was 10^10^ particles/mL, and serial dilutions (10^9^, 10^8^, and 10^7^ particles/mL) were subsequently evaluated for the capture events. Each experiment was performed in triplicate (*n* = 3), and the corresponding plasmonic responses were presented in Figure 5. As in previous capture studies, the same order and duration of three sequential steps were applied. The initiation of each phase was marked on the plasmonic graphs. For the 10^10^ particles/mL sample (Figure 5A–C), wavelength shifts of 1.79, 2.82, and 1.96 nm were recorded (average 2.20 ± 0.45 nm). At 10^9^ particles/mL (Figure 5D–F), the shifts were 1.23, 1.58, and 1.39 nm (average 1.40 ± 0.14 nm). When the concentration decreased to 10^8^ particles/mL, the red‐shifts of 0.94, 0.72, and 1.0 nm were observed (average 0.89 ± 0.12 nm) (Figure 5G–I). At the lowest tested concentration of 10^7^ particles/mL, the wavelength changed by 0.18, 0.25, and 0.15 nm (average 0.20 ± 0.04 nm) (Figure 5J–L). These responses were substantially higher than those previously obtained from 3‐MNHS‐ and APTES‐modified surfaces, confirming the enhanced binding efficiency of the thermoresponsive polymeric interface. Then, the averaged results were fitted using Pearson's correlation, yielding a strong linear relationship between EV concentration and wavelength shift (Figure 5M). The sensor demonstrated a dynamic detection range spanning approximately three orders of magnitude. Analysis of the linear calibration curve (Figure 5M) revealed a clear relationship between the wavelength shift (y) and EV concentration (x) over this range, described by the equation (y = 0.6518 log_10_ x–4.369). This correlation model was then used to calculate the mathematical limit of detection (LOD) (Equation 1). Here, *S* represents the standard deviation of the wavelength shift response, derived from the regression line intercept, and *b* is the slope. Based on these parameters, the LOD of the sensor was calculated as 1.6 × 10^7^ particles/mL.

(1) LOD=3S/b

Release experiments were performed using EV samples at 10^9^ particles/mL, corresponding to the intermediate range of concentrations tested in the experiments. Real‐time plasmonic responses across five independent capture–release cycles were shown in Figure S8. After capture and washing, the temperature was elevated from 27°C to 35°C—the transition point of PNIPAM—to induce release over 45 min at a flow rate of 10 µL/min. Following release, the temperature was lowered back to 27°C, and PBS was perfused for 10 min to stabilize the signal. The corresponding steps were as follows: (I) PBS baseline (5 min), (II) EV capture (45 min), (III) PBS washing (5 min), (IV) temperature increase and release event (45 min), and (V) temperature reduction (10 min). The wavelength shifts and calculated release efficiencies are summarized in Table S2, with recovery rates ranging from 18%–49% (mean: 36.35 ± 16.06%). No distinct pattern influencing release efficiency was evident, suggesting the dominance of local surface–polymer interactions in the desorption process.

On the other hand, surface blocking is a well‐established strategy to minimize non‐specific adsorption and improve selectivity, particularly in biofluids rich in proteins and macromolecules [33, 34, 35, 36]. In our system, PNIPAM provides an initial level of surface passivation by exposing its hydrophilic groups at temperatures below its LCST, forming a hydration layer that contributes to anti‐fouling properties [37, 38]. To further enhance passivation during EV release and improve system stability in biological environments, BSA was applied as an anti‐fouling agent. Beyond BSA, alternative strategies such as poly(ethylene glycol) (PEG)‐based coatings, zwitterionic polymers, or surfactant‐assisted blocking approaches could be implemented to further minimize non‐specific adsorption and surface fouling, particularly in complex biofluids [39, 40, 41].

To evaluate the influence of surface blocking on release performance, the metasurface sensors were treated with 5 mg/mL of BSA following PLL–PNIPAM–anti‐CD63 antibody modification. The sensors were incubated with BSA at 4°C for 20 min, rinsed thrice with PBS, and subjected to identical capture and release events. Compared with non‐blocked surfaces, BSA‐passivated sensors exhibited markedly improved release efficiency (Figure 5N). Across five replicates, release efficiencies ranged from 50.18% to 118.8% (mean: 87.03 ± 23.5%) (Table S3), indicating a substantial reduction in non‐specific binding events. The overall wavelength shifts during the capture phase were lower than those of unblocked surfaces, further confirming successful blocking. A single experiment exceeding full (100%) release efficiency likely stemmed from partial antibody detachment from the surface—a known limitation of immunoaffinity‐based isolation techniques [5]. Collectively, these results demonstrate that the PLL–PNIPAM–anti‐CD63 antibody–BSA modified plasmonic metasurface enables efficient and specific capture of EVs from the sensors within minimal sample volumes (∼5 µL). The captured EVs could be subsequently released through a mild temperature shift (27°C–35°C), exploiting the reversible hydrophilic–hydrophobic transition of PNIPAM without compromising vesicle integrity or surface protein structure.

Beyond the MCF‐7 model, we further demonstrated the broad applicability of our platform by targeting conserved tetraspanin markers (CD63) on EVs. The PNIPAM‐based metasurface is modular and can be readily adapted to capture EV subpopulations from diverse cellular origins. Using the same workflow established for MCF‐7‐derived CD63‐positive EVs, we successfully captured and released CD63‐positive EVs from HEK‐293 cells (Figure S9), confirming the generalizability of the platform across different cell lines.

### 3.8 Characterization of Released EVs

The primary goal of this study was to isolate EVs with high purity and decreasing heterogeneity of the sample while avoiding the use of harsh enzymatic or chemical treatments. To confirm the successful capture and release of intact EVs from the metasurface sensors, the collected samples were characterized using SEM, TEM, NTA, fNTA, and Western blotting, as described previously. EVs were initially collected using an ultrafiltration chip that separates vesicles based on their size via a dual‐filtration process [16]. Because our metasurface sensor isolates EVs through their surface tetraspanin marker (CD63), in addition to conventional NTA, we further quantified CD63‐positive EVs using fNTA. This enabled antigen‐specific enumeration of EVs, as demonstrated in the literature [42]. Again, the aforementioned MISEV criteria were strictly followed in this step [25].

**FIGURE 6:**
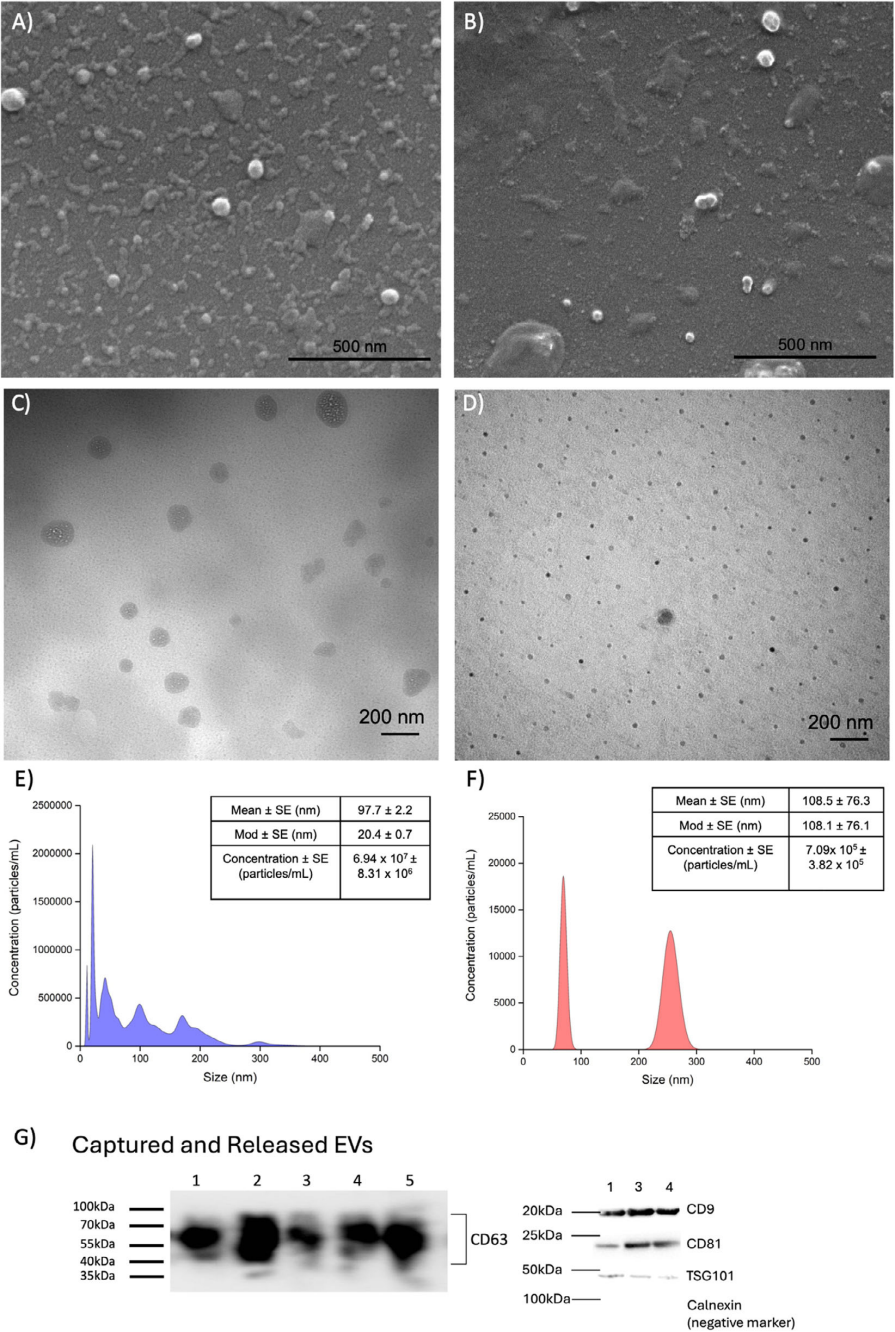
Characterization of EVs isolated after release events from PLL–PNIPAM–anti‐CD63 antibody–modified and BSA‐blocked sensors. SEM images of released EVs (A) at low and (B) at higher magnification, showing spherical morphology and integrity. Likewise, TEM images of (C) ultrafiltration‐isolated EVs prior to sensor detection and (D) sensor‐released (isolated) EVs following thermally induced desorption, both displaying intact, membrane‐bound vesicular morphology consistent with lipid bilayer–encapsulated particles. (E) Size distribution and concentration of isolated vesicles determined by NTA. (F) fNTA results of Alexa Fluor 488–conjugated anti‐CD63 antibody immunolabeled EVs, confirming tetraspanin‐positive vesicles. (G) Western blot analysis validating the presence of EV‐associated markers CD63, CD9, CD81, and TSG101 in the released EV samples, alongside the paucity of the endoplasmic reticulum marker calnexin, confirming vesicle identity and purity.

Similar to the ultrafiltration results, SEM imaging revealed the characteristic spherical morphology of the released EVs (Figure 6A,B). To provide higher‐resolution ultrastructural characterization, TEM imaging was additionally performed on both ultrafiltration‐isolated and sensor‐released (isolated) EV fractions (Figure 6C,D). TEM analysis confirmed the presence of intact, membrane‐bound vesicles with characteristic spherical morphology in both samples. The ultrafiltration‐isolated EVs displayed well‐preserved, rounded vesicles with clearly defined boundaries. Likewise, the sensor‐released EVs similarly retained their characteristic morphology with no evidence of membrane disruption attributable to the thermal release process. The reduced particle density observed in the released fraction is consistent with the inherently lower elution concentration following affinity‐based microfluidic isolation. While negative staining would further enhance membrane contrast and facilitate more detailed visualization of vesicular ultrastructure, its absence does not limit the validity of the present findings. The unstained TEM observations are consistent with NTA size distributions and biochemical marker analyses, collectively supporting the structural integrity and identity of the isolated vesicles. Incorporation of negative staining would further strengthen ultrastructural characterization in the future. Compared to the EVs isolated by ultrafiltration (Figure S6A,B), the number of EVs was lower; however, their size and morphology remained consistent, indicating that the thermoresponsive isolation process preserved vesicle integrity. NTA measurements were subsequently performed to determine the size distribution and concentration of the released EVs, with five independent measurements (*n* = 5), each recorded for 60 s. The EVs released from the sensors exhibited a mean diameter of 97.3 ± 2.2 nm and a modal value of 120.4 ± 0.7 nm, consistent with the typical EV size range (Figure 6E). Minor peaks were detected at higher diameters (299, 507, and 579 nm), which may potentially correspond to aggregates.

When comparing the two methods, the particle concentration measured by NTA was 1.01 × 10^10^ ± 5.23 × 10^8^ particles/mL for ultrafiltration and 6.94 × 10^7^ ± 6.31 × 10^6^ particles/mL for the sensor (Figure S6C,D). To further validate the EV‐specific capture, the released vesicles were immunostained with Alexa Fluor 488–labeled anti‐CD63 antibodies and analyzed using fNTA under a blue laser excitation source. The mean and modal sizes were measured as 108.5 ± 7.6 and 108.1 ± 7.6 nm, respectively (Figure 6F). In the fNTA measurements, the disappearance of the higher‐size peaks (around 500 nm) suggested the removal of non‐vesicular contaminants such as salt crystals or dust particles by utilizing the sensors. A minor shift in size distribution toward 200–300 nm was observed, which may result from partial detachment of surface‐bound antibodies during the thermoresponsive release process [5].

Comparatively, the concentration of fluorescently‐labeled EVs determined by fNTA was 1.98 × 10^6^ ± 1.71 × 10^5^ particles/mL for ultrafiltration and 7.09 × 10^5^± 3.82 × 10^5^ particles/mL for the metasurface sensor (Figure 6F). Moreover, an enrichment factor (Equation (2)) was defined to quantify the degree to which the metasurface sensor selectively isolated bona fide EVs, thereby reflecting the improvement in sample purity over the conventional size‐based ultrafiltration method:

(2) $$\mathrm{Enrichment}\;\mathrm{Factor}=\frac{\left(\frac{\mathrm{Number}\;\mathrm{of}\;\mathrm{tagged}\;\mathrm{EVs}\;\left(\mathrm{sensor}\right)}{\mathrm{Number}\;\mathrm{of}\;\mathrm{untagged}\;\mathrm{EVs}\;\left(\mathrm{sensor}\right)}\right)}{\left(\frac{\mathrm{Number}\;\mathrm{of}\;\mathrm{tagged}\;\mathrm{EVs}\;\left(\mathrm{ultrafiltration}\right)}{\mathrm{Number}\;\mathrm{of}\;\mathrm{untagged}\;\mathrm{EVs}\;\left(\mathrm{ultrafiltration}\right)}\right)}$$

According to Equation (2), the thermoresponsive metasurface sensor provided up to 100‐fold improvement in EV sample purity, attributed to selective isolation via CD63 recognition, which significantly reduced non‐vesicular contaminants and debris. Given the inherent heterogeneity of EVs in size, surface composition, and protein content, our strategy with affinity‐based thermoresponsive capture and release—substantially enhanced sample specificity and uniformity.

Lastly, Western blot analysis was employed to confirm the molecular identity of the released EVs and the presence of the CD63 tetraspanin protein on their surface (Figure 6G). EV samples captured and released from BSA‐blocked plasmonic metasurfaces (five replicates) were collected for analysis. CD63 is known to undergo post‐translational *N*‐glycosylation modifications [43, 44], often resulting in a diffuse band pattern between 35 and 80 kDa. The Western blot results clearly confirmed the presence of CD63 within the collected EV samples, verifying that the tetraspanin marker remained intact after the capture and release events. In addition to CD63, another set of Western blot analysis was carried out to confirm the presence of CD9, CD81, and TSG101—canonical EV‐associated markers consistent with EV identity according to MISEV2018 guidelines. Notably, calnexin, an endoplasmic reticulum–resident protein serving as a negative control, was absent in all released EV fractions, confirming the absence of cellular organelle contamination and supporting the purity of the isolated vesicles. In addition to the protein validation, RNA cargo analysis would provide complementary insight into EV content. However, it is not required under the MISEV2023 minimal criteria for EV characterization. Since we demonstrate a proof‐of‐concept strategy in this study, we closely follow and fulfill both MISEV2018 and MISEV2023 guidelines as a minimum requirement for EV characterization [5]. The multi‐marker protein validation presented here fully satisfies these standards, supporting the identity and integrity of the isolated vesicles. RNA‐based analyses would also be an important direction for future studies as the platform is further developed. Collectively, SEM, NTA, fNTA, TEM, and Western blot analyses corroborate that the PNIPAM‐modified metasurface platform enables the gentle and efficient isolation of EVs while preserving their structural and biochemical integrity. The absence of harsh chemicals or enzymatic treatments, coupled with high purification performance, underscores the promise of this thermoresponsive plasmonic system for label‐free and minimally invasive EV separation applications.

## 4 Conclusions

This work introduces a thermoresponsive plasmonic metasurface sensor as a versatile and label‐free platform for the controlled capture and on‐demand release of EVs. By integrating PNIPAM onto nanograted metasurfaces functionalized with anti‐CD63 antibodies, the system leverages temperature‐induced hydrophilic–hydrophobic transitions to enable reversible EV binding without chemical disruption. Using MCF‐7–derived EVs as a model system, the platform achieved real‐time monitoring across a dynamic detection range of 10^7^–10^10^ particles/mL, with a detection limit of 1.6 × 10^7^ particles/mL and release efficiency of 87.03 ± 23.5% following surface passivation with BSA. Complementary analyses—including SEM, TEM, NTA, fNTA, and Western blotting—confirmed the high purity (up to ∼100‐fold increase in purity compared to the ultrafiltration process), preserved morphology, and biomarker integrity of the released vesicles. Additionally, protein levels in EVs may fluctuate slightly following exposure to 37°C, but no drastic changes are expected since this temperature is within the physiological range [45, 46]. Certain cell types may be more sensitive to such temperature shifts, which should be taken into account when designing experiments, although our NTA, SEM, TEM, and Western blot data indicate that EV integrity is maintained.

In addition to our approach (microfluidic ultrafiltration), as the other set of experiments, we performed comparative EV isolation using ultracentrifugation (UC) and immunomagnetic bead‐based methods (MB). Overall, all methods yielded comparable EV concentrations, with no statistically significant differences observed, but exhibited distinct size distributions. UC produced a particle concentration of 5.26 × 10^8^ ± 2.18 × 10^7^ particles/mL with a narrow, unimodal size distribution centered at 110.6 ± 2.8 nm, consistent with its established efficiency in isolating small EVs. However, UC requires specialized high‐speed instrumentation, extended processing times, and may co‐isolate protein aggregates and non‐vesicular contaminants, limiting its practicality in resource‐limited or point‐of‐care settings. Our method yielded 3.07 × 10^8^ ± 2.34 × 10^7^ particles/mL with a modal size of 139.6 ± 11.9 nm, representing a more accessible and reproducible alternative with reduced equipment demands. MB isolation produced a comparable concentration (2.97 × 10^8^ ± 1.46 × 10^7^ particles/mL), but exhibited a broader and more heterogeneous size distribution, including a secondary peak at approximately 280 nm. This likely reflects bead‐associated aggregates or non‐specific binding, consistent with known limitations of bead‐based approaches. Collectively, these results highlight the advantage of our platform in achieving reliable and specific EV isolation without reliance on ultracentrifugation infrastructure or complex bead‐based workflows, making it particularly well‐suited for miniaturized and point‐of‐care diagnostic applications (Figure S10).

This proof‐of‐concept platform offers several advantages over conventional EV isolation systems, including portability (1.5 cm × 1.5 cm for a sensor and 9 cm × 12 cm for the readout device), low‐cost ($1.50 per sensor), repeatable measurements, and compatibility with low‐volume samples (300‐450 µL). The hand‐held setup eliminates the need for complex optical configurations or training, making it suitable for point‐of‐care diagnostics. Still, several improvements are required before clinical translation. First, surface blocking remains a critical factor for achieving higher selectivity and reproducibility; optimizing the surface chemistry with agents such as PEG, zwitterionic polymers, or surfactant‐assisted blocking strategies could further minimize nonspecific adsorption. Although this study focused on CD63‐positive EVs from MCF‐7 and HEK‐293 cells, the PNIPAM‐based metasurface is highly versatile and can be readily adapted to capture other EV subpopulations using alternative markers such as CD9, CD81, EpCAM, and HER2. This flexibility is enabled by the robust succinimide–NH_2_ surface chemistry, which allows straightforward functionalization with diverse antibodies, supporting targeted isolation of multiple EV markers and subpopulations. Second, the limited area on metasurface sensors hinders capture efficiency, eventually leading to reduced particle concentrations after release. This can be addressed by expanding the channel geometry or increasing the active surface area to enhance binding capacity. Additionally, the localized plasmonic field strength decays rapidly with distance from the surface, necessitating optimized molecular spacing for efficient signal transduction. Overall, this thermoresponsive metasurface‐based sensing system demonstrates a robust framework for selective and spatiotemporal EV isolation and real‐time analysis. With continued refinement in surface engineering and fluidic integration, this technology holds strong potential as a next‐generation point‐of‐care tool for EV‐based diagnostics, precision oncology, and personalized medicine.

## Conflicts of Interest

The authors declare no conflict of interest.

## Supporting information




**Supporting File**: adma73093‐sup‐0001‐SuppMat.pdf.

## Data Availability

The data that support the findings of this study are available from the corresponding author upon reasonable request.
