# Supplementary material for: Thermally Modulated Metasurface Sensor for Dynamic and Time‐Resolved Isolation of Extracellular Vesicles
Source: Adv Mater. 2026 May 8;38(44):e22964. doi: 10.1002/adma.202522964 (PMC13449105; doi:10.1002/adma.202522964)
Supplement: Supplementary file 1 [file ADMA-38-e22964-s001.pdf]

Supporting Information

Thermally Modulated Metasurface Sensor for Dynamic and Time-Resolved Isolation of Extracellular Vesicles

Beyza Nur Kucuk[1,2], Eylul Gulsen Yilmaz[1,2], Yusuf Aslan[1,2], Emre Ece[1,2], Özgecan Erdem[1], Murat Alp Gungen[1,2], Bengi Özgün Öztürk[3], Fatih Inci[1,2*]

[1]UNAM—National Nanotechnology Research Center, Bilkent University, 06800, Ankara, Turkey

[2]Institute of Materials Science and Nanotechnology, Bilkent University, 06800, Ankara, Turkey

[3]Hacettepe University, Faculty of Science, Department of Chemistry, 06800, Ankara, Türkiye

*Corresponding author
E-mail: finci@bilkent.edu.tr

Table of Content

Experimental Section…………………………………………………………………...3

Contact Angle ………………………………………………………………….....3

X-ray photoelectron Spectroscopy (XPS)……………………………………......3

Surface Morphology Analysis using Keyence VK X100 and SEM ……………..3

Nanoparticle Tracking Analysis (NTA) ……………………………………………3

SEM and TEM for EV Morphology ………………………………….……..4

Western Blot Analysis …………………………………………………………4

Surface Chemistry Optimization ……………………………………….......4

HEK293 Cell Culture…………………………………………………………5

Comparative EV Isolation Methods………………………………………...5

Supplementary Figures ………………………………………………………6

Figure S1 ……………………………………………………………………...6

Figure S2 ……………………………………………………………………...7

Figure S3 ……………………………………………………………………...8

Figure S4 ……………………………………………………………………...9

Figure S5 ……………………………………………………………………10

Figure S6 ……………………………………………………………………11

Figure S7 ……………………………………………………………………12

Figure S8 ……………………………………………………………………13

Figure S9……………………………………………………………………..14

Figure S10…………………………………………………………………....15

Table S1 ……………………………………………………………………...16

Table S2 ……………………………………………………………………...17

Table S3 ……………………………………………………………………...18

Experimental Section

Contact Angle

The wettability of (i) bare metasurfaces, (ii) PLL-modified surfaces, (iii) PLL + PNIPAM-modified surfaces, and (iv) PLL + PNIPAM + antibody-modified surfaces was evaluated by contact angle measurements using a Dataphysics OCA 30 system (Filderstadt, Germany). For each sample, 5 µL of ultrapure water was dropped on the surface by the sessile drop method, and the contact angle was recorded immediately. Measurements were repeated three times, and results were reported as mean ± standard deviation.

X-ray photoelectron Spectroscopy (XPS)

Surface modifications were characterized by XPS (K-Alpha, Thermo Scientific, USA). Samples analyzed included (i) bare metasurface, (ii) PLL-modified surfaces, (iii) PLL + PNIPAM-modified surfaces, and (iv) PLL + PNIPAM + antibody-modified surfaces. A complete survey spectrum was acquired, and elemental peaks of carbon (C), nitrogen (N), and gold (Au) were examined. Data were referenced to the C1s peak and normalized using OriginPro.

Surface Morphology Analysis using Keyence VK X100 and SEM

Surface morphology of the metasurfaces was examined by SEM (Quanta 200 FEG, FEI, USA) for (i) bare metasurfaces, (ii) PLL-modified surfaces, and (iii) PLL + PNIPAM-modified surfaces. Samples (0.5 × 0.5 mm) were mounted on carbon tape and imaged under high vacuum with a working distance of 10 mm. In addition, PLL + PNIPAM-modified surfaces were analyzed using a laser scanning microscope (Keyence VKX-100, Keyence, Japan) with 50× magnification and a scanning thickness of 1 µm to observe the PNIPAM coating.

Nanoparticle Tracking Analysis (NTA)

EV size and concentration were analyzed by nanoparticle tracking analysis (NTA, NS300, Malvern Instruments Ltd., UK). EV samples were diluted 1:50 in DPBS (0.22 µm filtered) and introduced into the NTA chamber. Measurements were performed using a 488 nm laser, with viscosity set to 0.92. For each run, five 60-s videos were recorded, and 0.2 mL of fresh sample was loaded between captures.

SEM and TEM for EV Morphology

The morphology of EVs was examined by SEM (Quanta 200 FEG, FEI, USA). Silicon wafers (0.5 × 0.5 mm) were sterilized by sonication in 70% ethanol for 5 min, dried, and mounted on carbon tape. EV samples were mixed with 4% paraformaldehyde (PFA) (1:1, v:v) for fixation, and 5 µL of the mixture was dropped onto the wafers and dried for 2 h. The samples were rinsed three times with ethanol, completely dried, coated with a 10 nm Au–Pd layer, and imaged under high vacuum. For TEM (JEOL JEM 100CX II, Tokyo, Japan) analysis, EV samples were mixed with 4% paraformaldehyde solution at a 1:1 (v/v) ratio for fixation. Fixed samples were adsorbed onto carbon-coated copper grids and allowed to settle at room temperature until completely dry prior to imaging.

Western Blot Analysis

Western blotting was used to confirm EV protein markers. EV samples were mixed with loading dye, denatured at 95 °C for 5 min, and run on SDS-PAGE. Proteins were transferred to PVDF membranes and blocked with 5% milk-TBST. Membranes were incubated overnight at 4 °C with anti-CD63 primary antibody (1:500, Recombinant Rabbit monoclonal antibody, Invitrogen MA5-30187, ThermoFisher), followed by HRP-conjugated goat-anti-rabbit secondary antibody (1:5000). Detection was performed using enhanced chemiluminescence (ECL, Luminol/Enhancer and Stable Peroxide Buffer (1:1, v:v)) and visualized with the ChemiDoc MP imaging system (Bio-Rad, California, USA).

Surface Chemistry Optimization

Alternative surface chemistries were explored for EV capture. Two strategies were tested: (i) 3-MNHS, a thiol-NHS crosslinker forming self-assembled monolayers on gold; and (ii) APTES, a silane reagent presenting amines for NHS–PNIPAM conjugation. EV samples (10^9 EVs/mL after dilution) were flowed at 10 µL/min with standard cycles of PBS (5 min) (I), EV solution (45 min) (II), and PBS wash (5 min) (III). For 3-MNHS, wavelength shifts after washing were 0.18 nm and 0.20 nm (0.19 ± 0.1 nm) (Fig. S8A-B). For APTES, shifts were 0.01 nm and 0.32 nm (0.165 ± 0.155 nm) (Fig. S8C-D). Both chemistries produced signals lower than the background noise of the microfluidic system. Therefore, PLL–PNIPAM functionalization was chosen as the most reliable modification strategy for EV capture and release.

HEK293 Cell Culture

The human embryonic kidney cell line HEK293 was maintained in Dulbecco's Modified Eagle Medium/Nutrient Mixture F-12 (DMEM/F-12) supplemented with 10% fetal bovine serum (FBS) and 1% penicillin–streptomycin. Cells were grown in T75 culture flasks under standard conditions at 37 °C in a humidified atmosphere containing 5% CO_2. The growth medium was replaced every two days, and cells were passaged at 80–90% confluency by detachment with 0.25% trypsin–EDTA solution.

Comparative EV Isolation Methods

For ultracentrifugation, conditioned medium was harvested from MCF-7 cells cultured in T225 flasks on day 4. A 5 mL aliquot was first centrifuged at 20,000 × g for 15 min at room temperature to sediment cell debris and large particles, after which the supernatant was passed through a 0.22 μm syringe filter. The clarified filtrate was then subjected to ultracentrifugation at 100,000 × g for 70 min at 4 °C using a P50AT2 rotor. The resulting pellet was carefully resuspended in PBS and reserved for downstream characterization.

For immunomagnetic bead-based isolation, 5 μL of capture beads were combined with 500 μL of conditioned medium in round-bottom microtubes. Following gentle vortexing, the mixture was incubated overnight at room temperature in the dark to allow EV binding. Beads were subsequently collected using a magnetic rack, and the supernatant was aspirated without disturbing the bead pellet. To confirm EV capture, 5 μL of CD81–PE detection antibody was introduced and incubated for 60 min at 4 °C in the dark. After a final wash with 1× Assay Buffer, the bead pellet was resuspended in 350 μL of assay buffer and analyzed by flow cytometry within 2 hours.

For microfluidic ultrafiltration, the same volume of conditioned medium was processed using the custom-designed chip as described in the main text.

Supplement figures

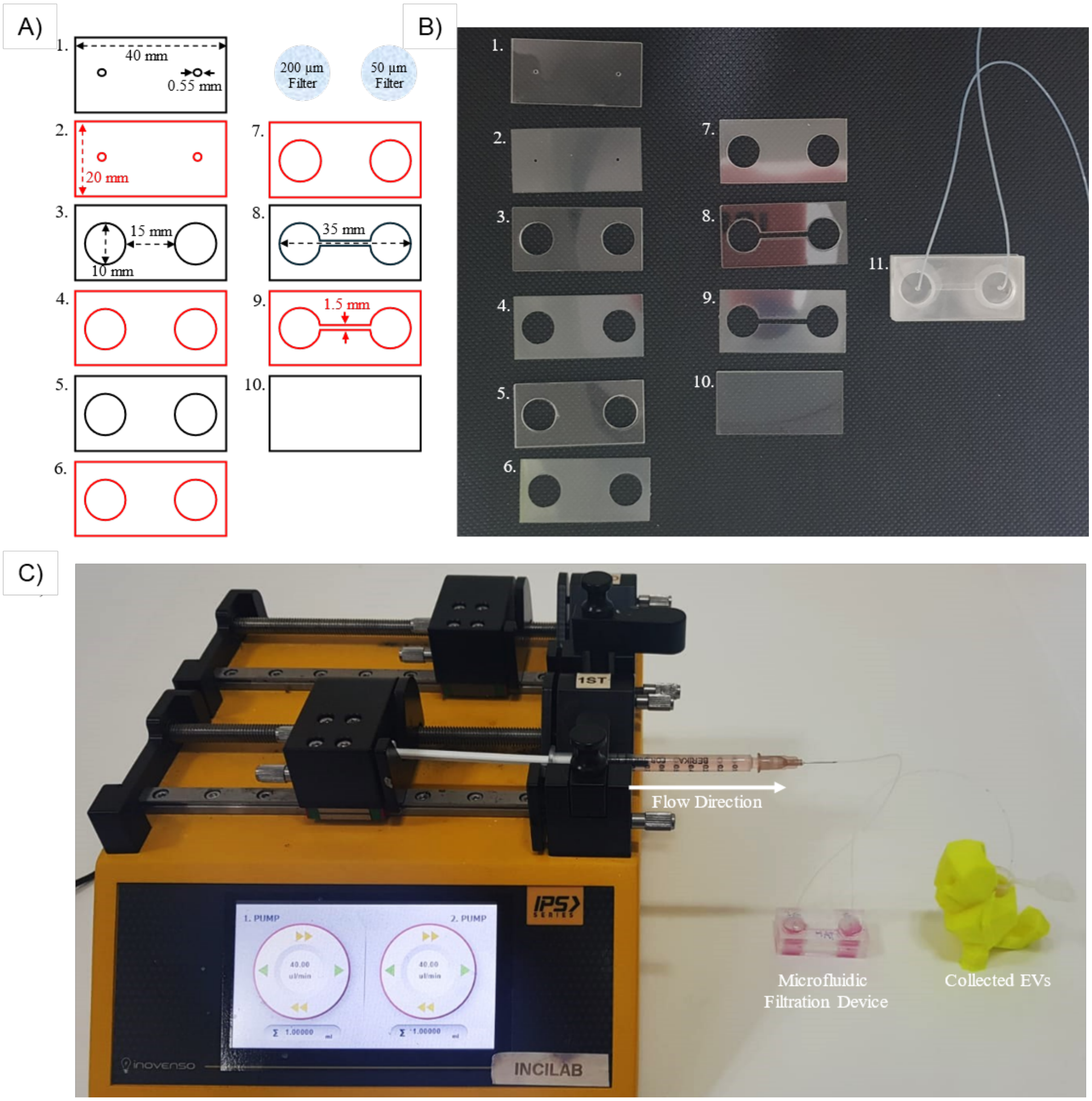

Figure S1. A) PMMA and DSA layers of the microfluidic chip were shown, where the black regions represent PMMA designs (thickness: 2 mm) and the red regions indicate DSA layers (thickness: 50 µm), with polycarbonate filters of 200 and 50 µm pore sizes placed between DSA layers 6 and 7. B) Laser-cut PMMA and DSA layers and the completed filtration chip with tubings (number 11) were presented. C) The finalized size-based EV isolation system, consisting of the microfluidic chip and syringe pump, was depicted.

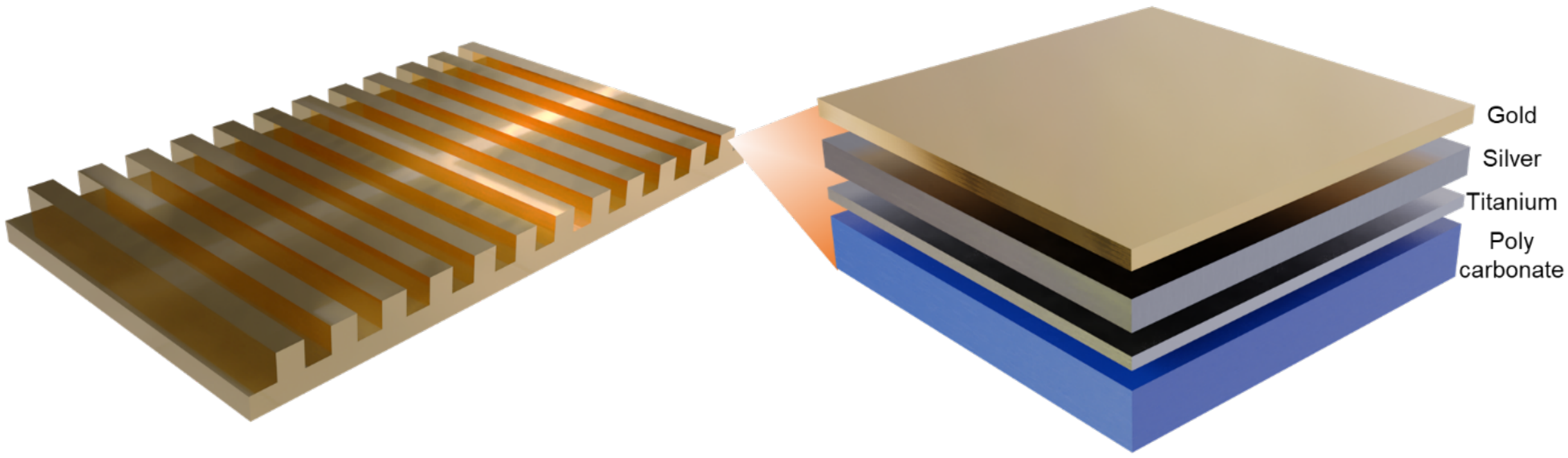

Figure S2. Illustration of metasurface and the layers.

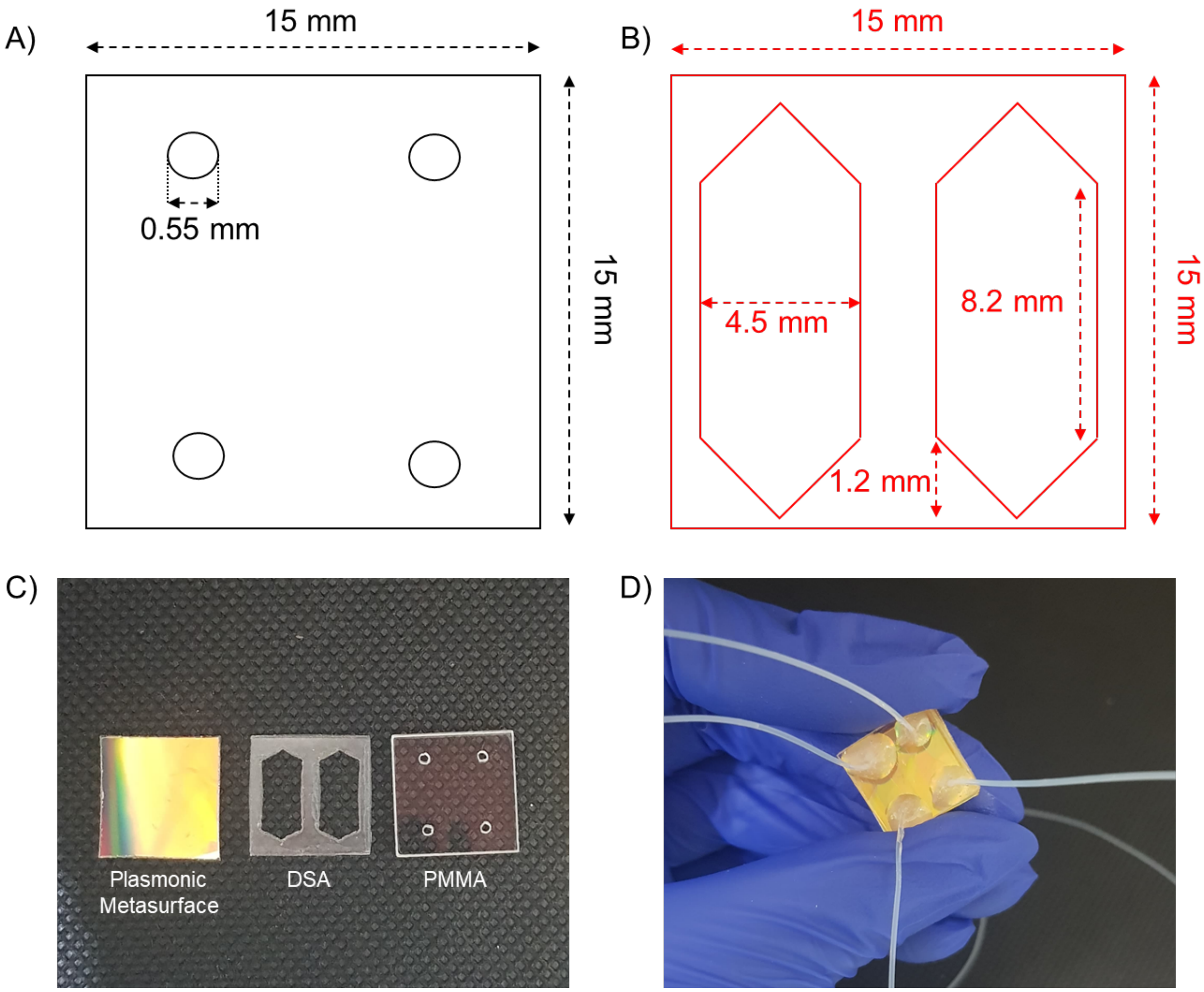

Figure S3. Metasurface-integrated microfluidic chip preparation was illustrated, including A) the PMMA layer with its dimensions, B) the DSA layer with its dimensions, C) the plasmonic metasurface, DSA, and PMMA layers shown separately, and D) the fully assembled microfluidic chip with capillary tubings.

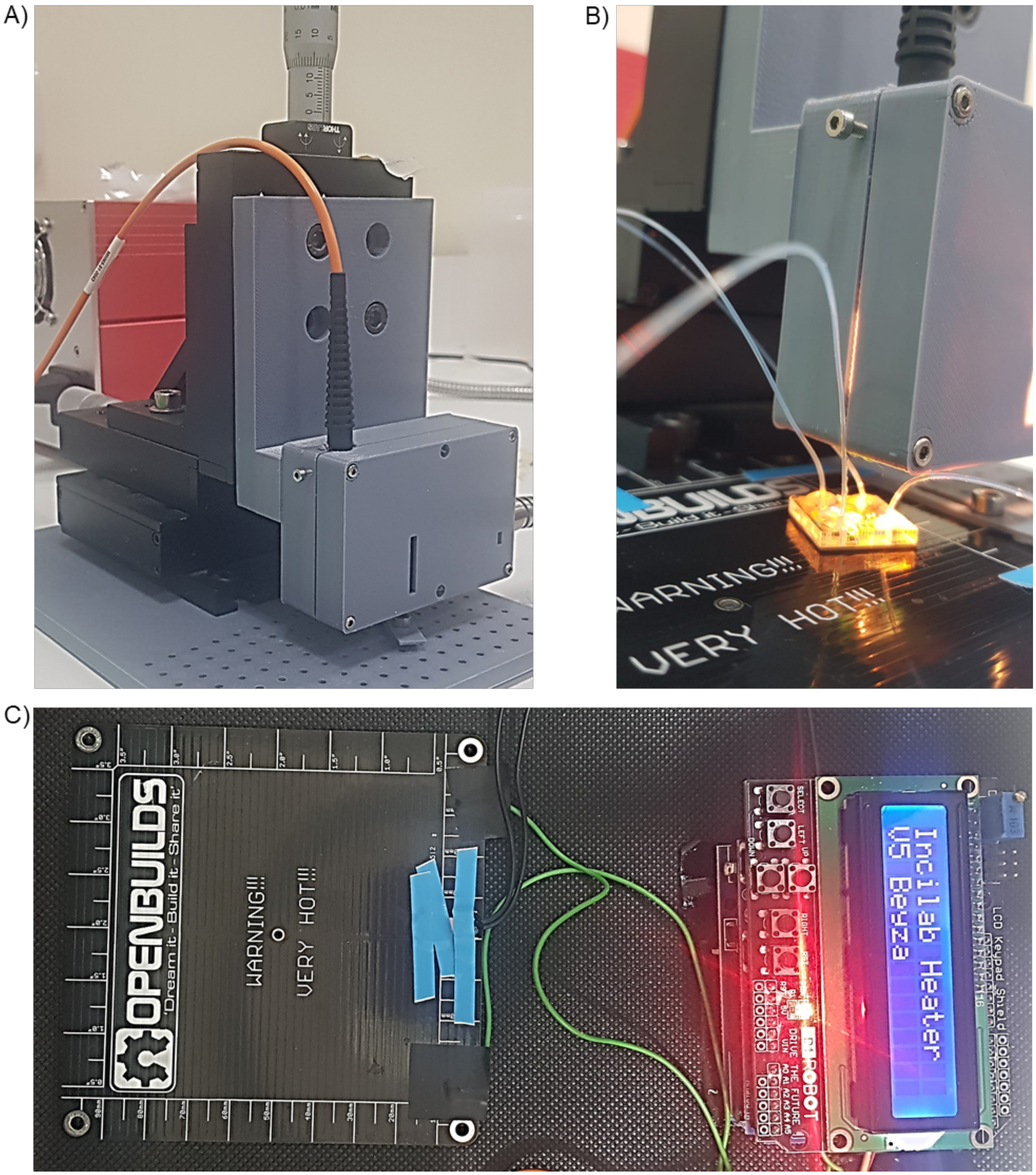

Figure S4. A) The portable surface polarization measurement device, B) the PCB heater with microfluidic plasmonic metasurface real-time data analysis, and C) the Arduino-assisted PCB heater for PNIPAM phase transition were depicted.

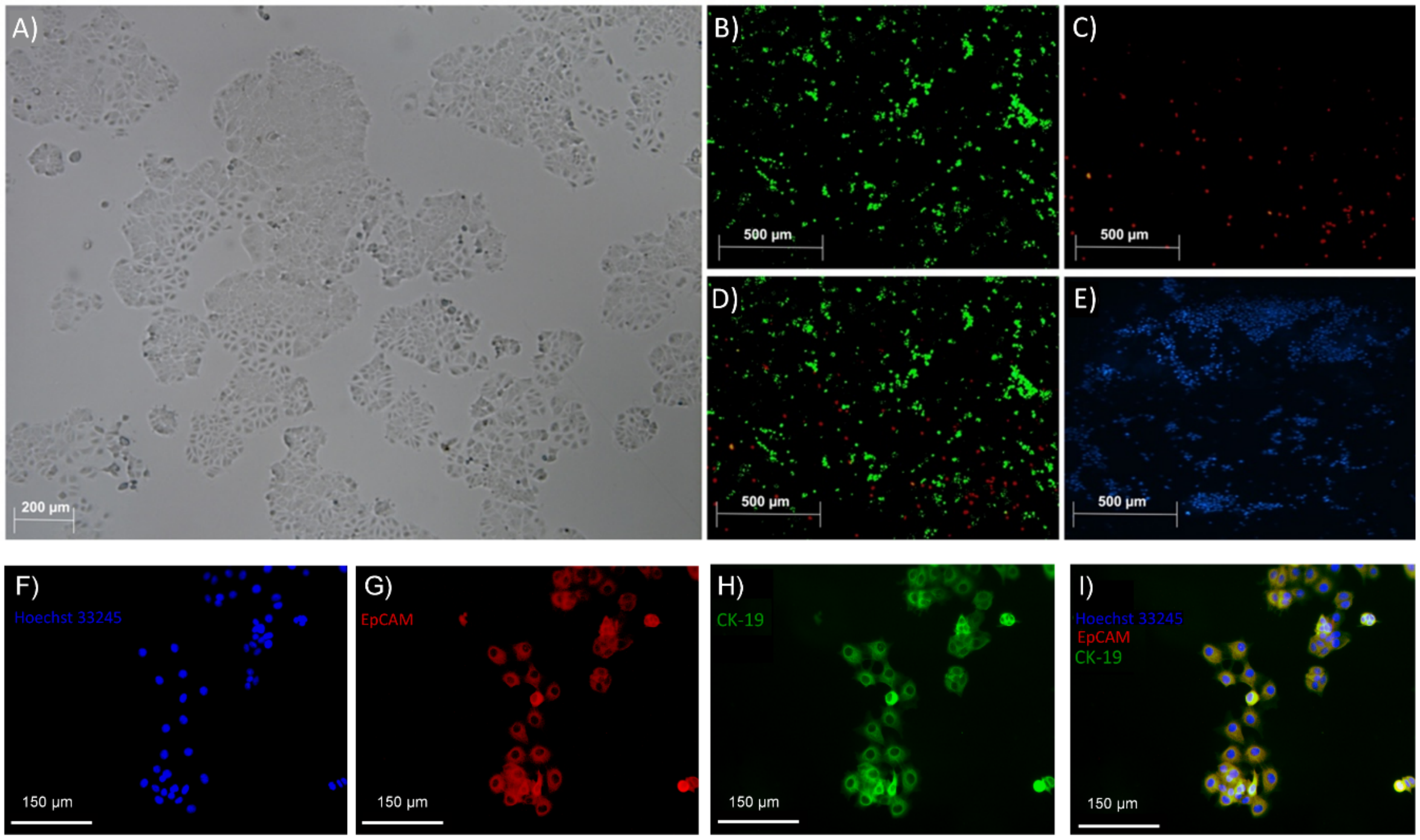

Figure S5. Microscopic characterization of cultured MCF-7 cells. A) Bright-field image of adherent MCF-7 cells. B) Live cells stained with Calcein-AM visualized under the GFP filter. C) Dead cells stained with ethidium homodimer observed using the RFP filter. D) Merged fluorescence image showing live/dead staining. E) Hoechst 33342–stained nuclei visualized under the DAPI filter. F) Hoechst 33342 staining confirming nuclear localization in immunohistochemistry (IHC) analysis. G) Alexa Fluor 647–labeled EpCAM expression indicating epithelial cancer biomarkers. H) FITC-labeled CK-19 expression representing epithelial cytokeratin biomarkers. I) Merged fluorescence image combining all channels, acquired using the fluorescence microscope.

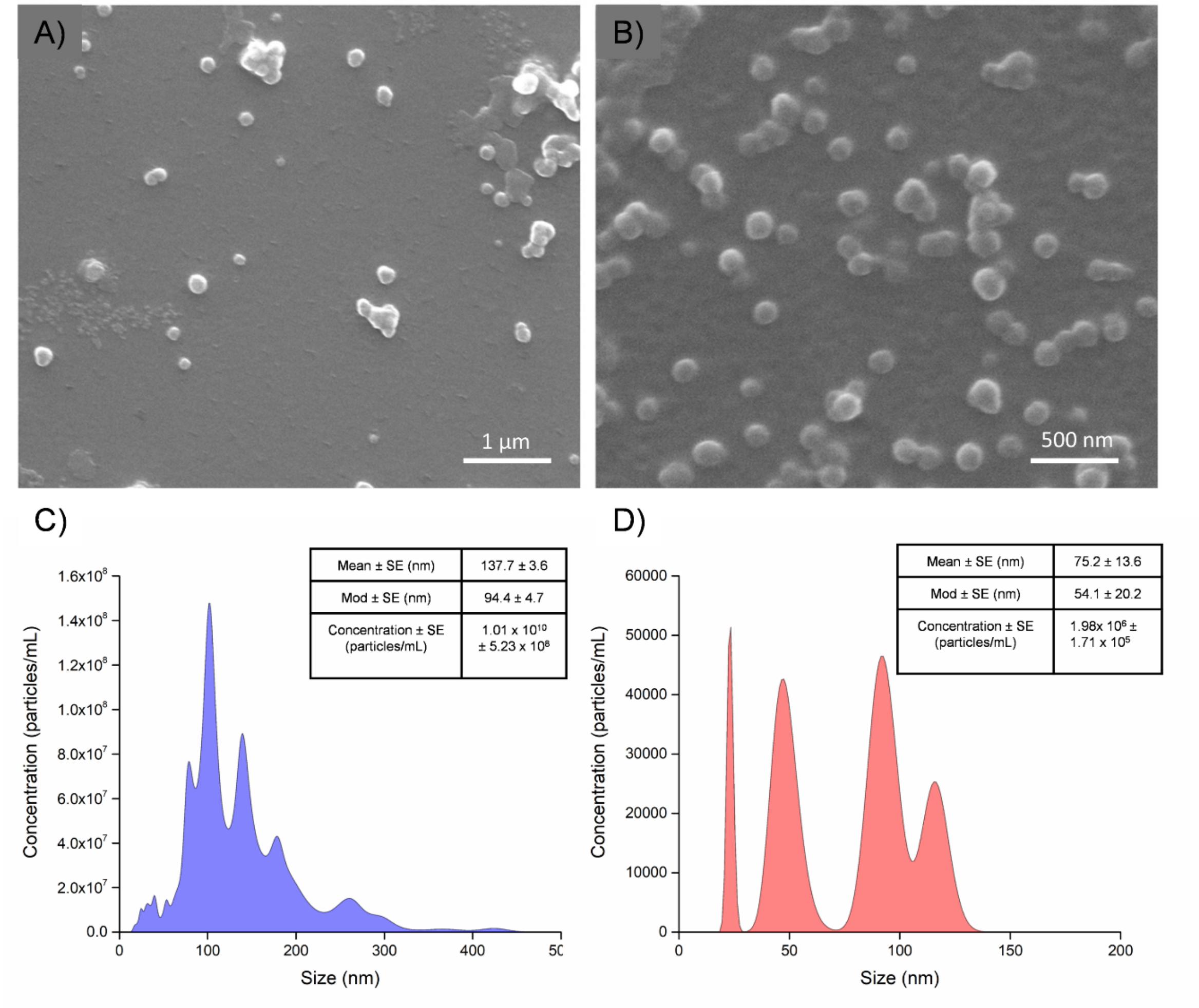

Figure S6. Characterization of EVs isolated *via* microfluidic ultrafiltration. A–B) SEM images depicting the morphology of isolated EVs at different magnifications. C) Size distribution and concentration of EVs determined by NTA. D) fNTA of Alexa Fluor 488–conjugated anti-CD63 immunolabeled EVs, confirming the selective detection of tetraspanin-positive vesicles.

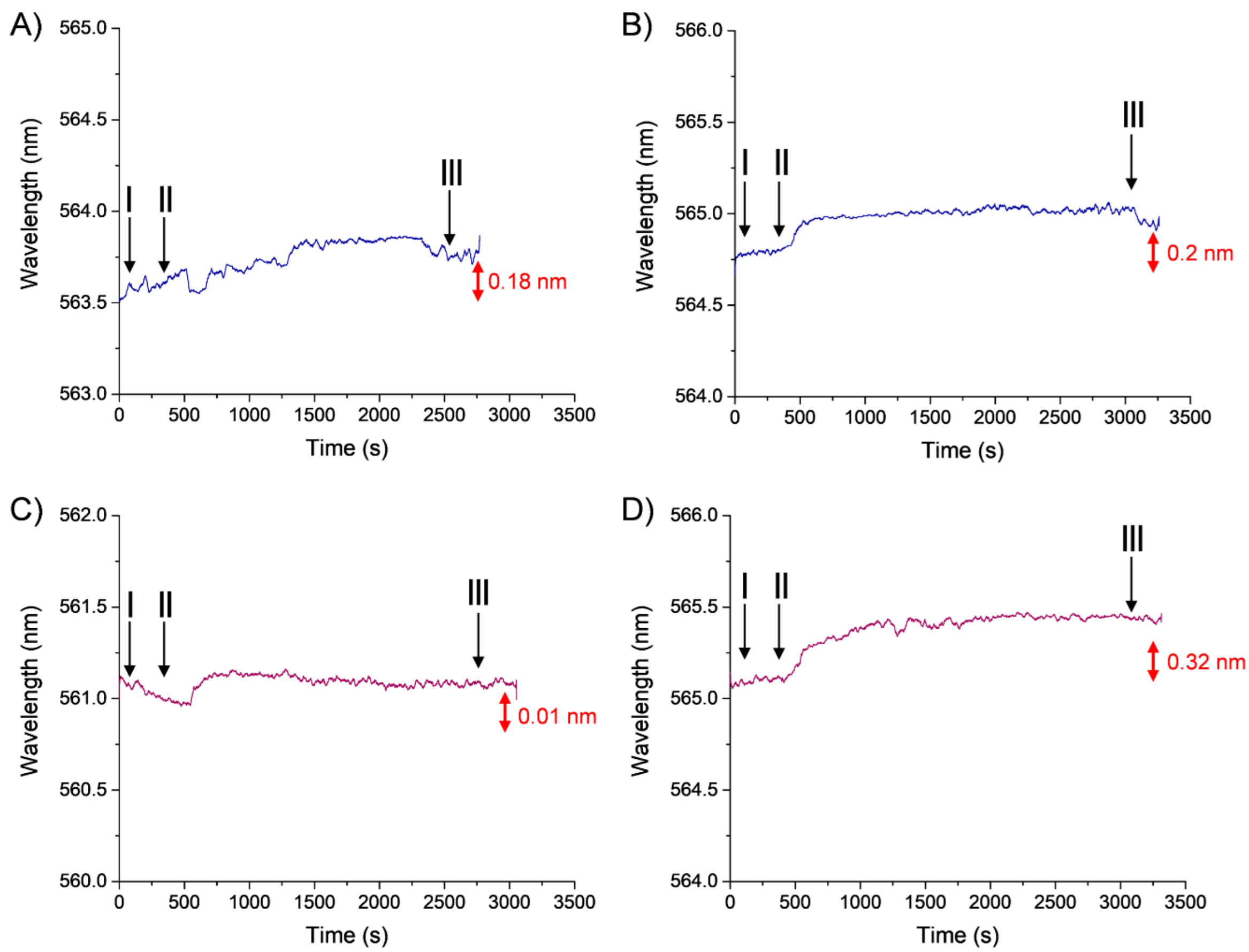

Figure S7. Optimization of surface chemistry on the sensors for EV capture at 10^9 particles/mL. A–B) Sensors modified with 3-MNHS, PNIPAM, and anti-CD63 antibody. C–D) Sensors decorated with APTES, PNIPAM, and anti-CD63 antibody.

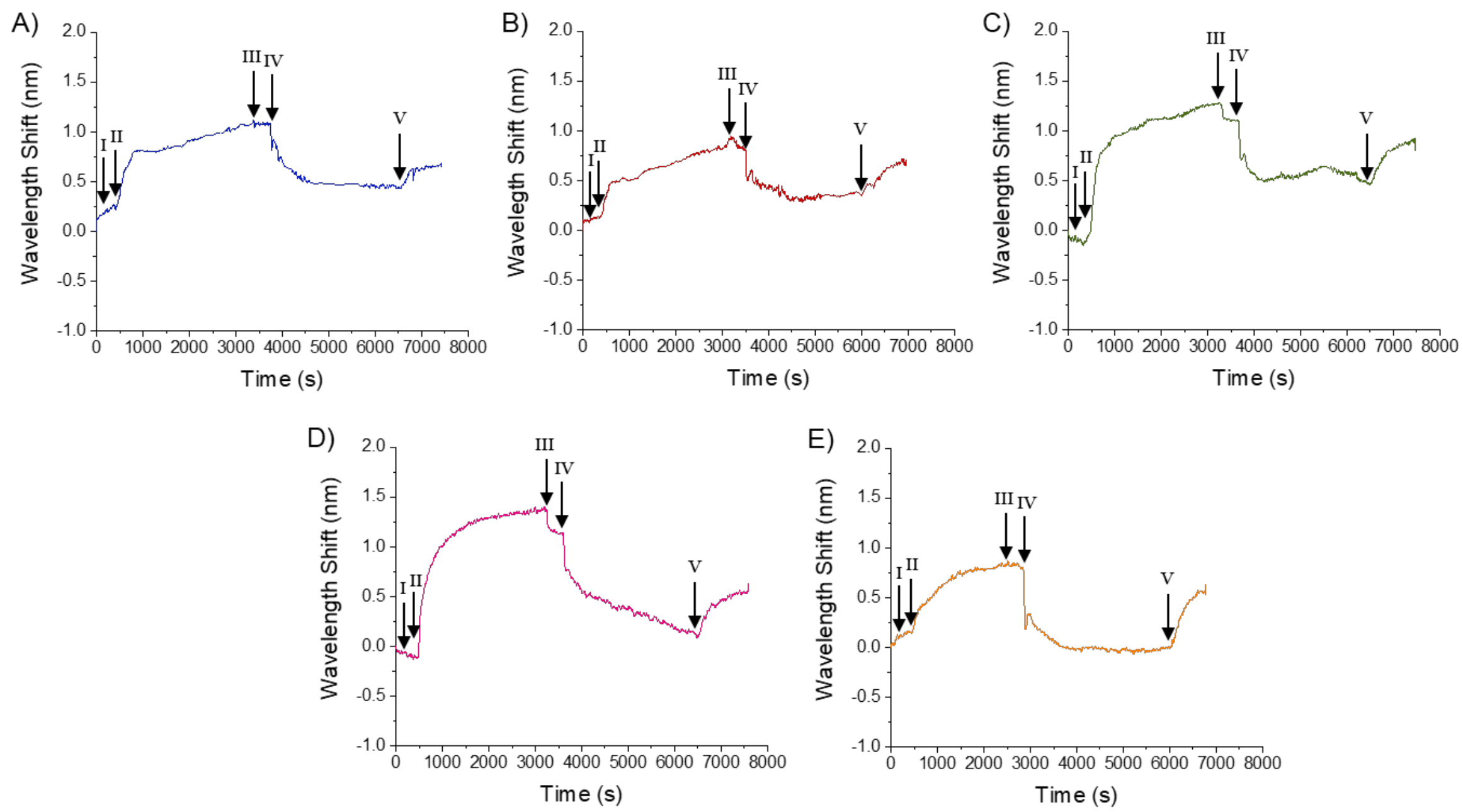

Figure S8. Real-time plasmonic monitoring of EV capture and release events on PLL–PNIPAM–anti-CD63 antibody–modified sensors without surface blocking (n=5).

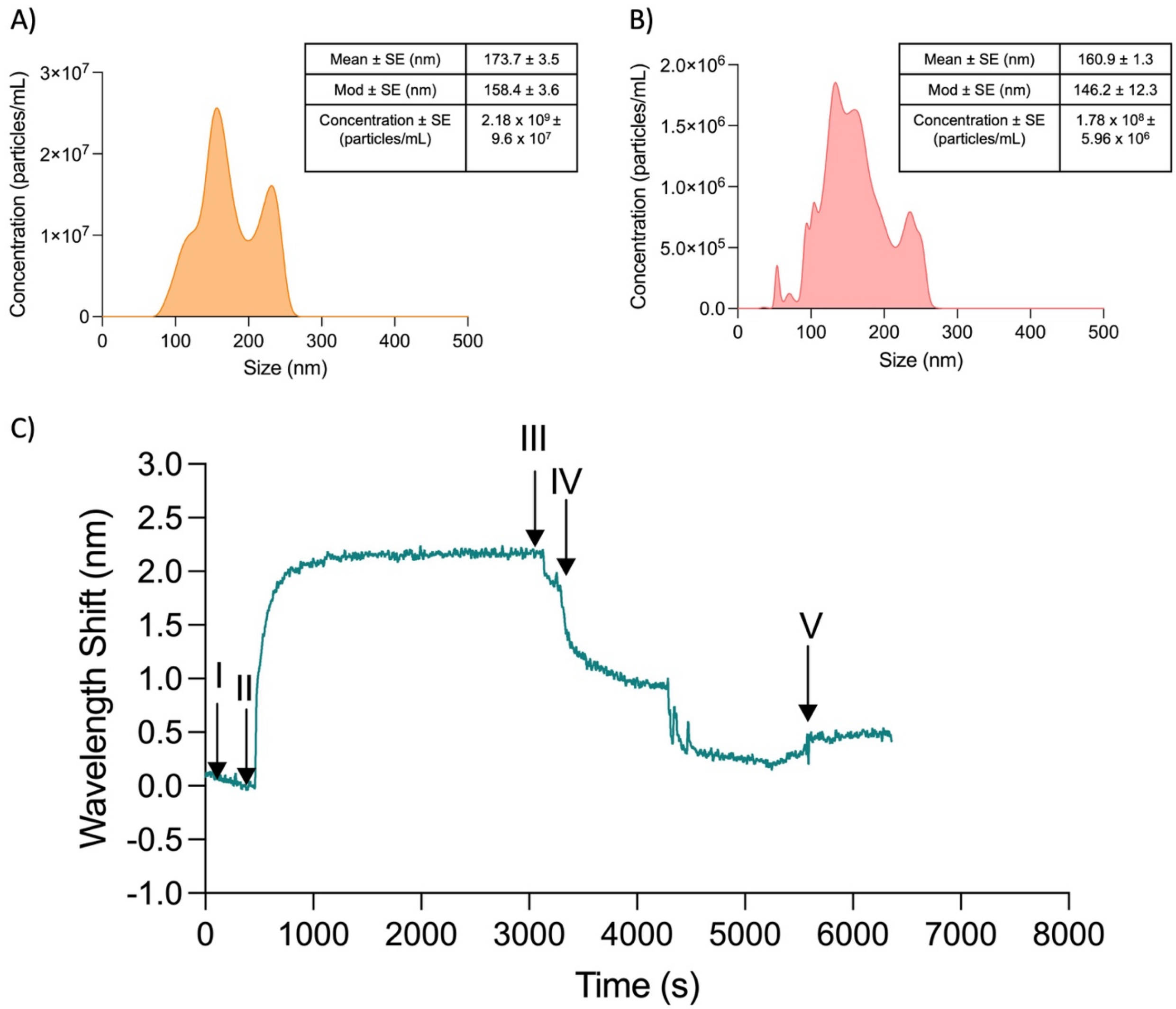

Figure S9. Characterization of HEK293-derived EVs and real-time plasmonic monitoring of capture and release events on PLL–PNIPAM–anti-CD63 antibody–BSA modified sensors. A) NTA size distribution of EVs prior to sensor loading. B) NTA size distribution of EVs following thermally induced release from the sensor surface. C) Real-time plasmonic response during capture and release events.

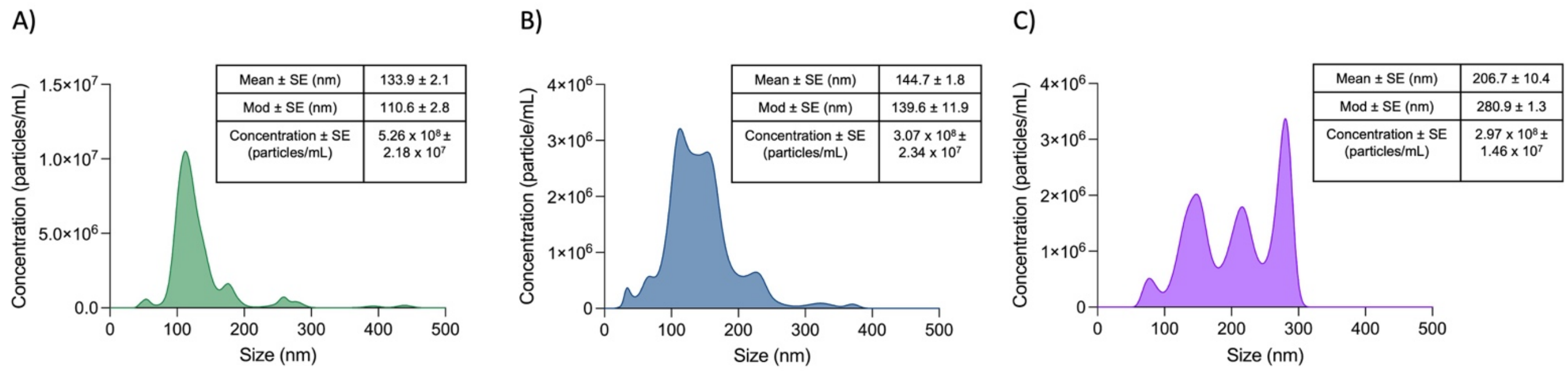

Figure S10. Comparative NTA analysis of MCF-7-derived EVs isolated by three different methods from identical starting volumes (5 mL conditioned medium, day 4). A) Ultracentrifugation (UC), B) microfluidic ultrafiltration (UF), and C) immunomagnetic bead-based isolation (MB). Size distributions and corresponding particle concentrations are shown for each method.

Table S1. ^{1}H NMR Chemical Shifts and Corresponding Bond Assignments of PNIPAM.

Peak Label	Peak Shift (ppm)	Origin
a	~7.46	Amide proton of PNIPAM (-NH)
b	3.95 – 3.44	Backbone methine proton (-CH)
c	1.82 – 1.06	Methyl groups (CH_3)
d	1.5 – 2.0	Methine proton (-CH)
e	0.9 – 1.2	Methylene groups (-CH_2) and succinimide terminal protons
-	3.3 – 3.9	Residual water
-	2.5	DMSO

Table S2. Wavelength shifts and release efficiencies of release events without surface blocking

Experiment	Wavelength Increase (Capture) (nm)	Wavelength Decrease (Release) (nm)	Release Efficiency (%)
A	0.85	0.42	49.05
B	0.73	0.20	27.62
C	1.23	0.22	18.14
D	1.22	0.60	48.81
E	0.70	0.27	38.15

Table S3. Wavelength shifts and release efficiencies of release events with surface blocking

Experiment	Wavelength Increase (Capture) (nm)	Wavelength Decrease (Release) (nm)	Release Efficiency (%)
i	0.44	0.43	98.84
ii	0.44	0.53	118.80
iii	0.45	0.43	94.44
iv	0.40	0.29	72.93
v	0.40	0.20	50.18
